# Tasmannioside A, a New Eudesmane Sesquiterpene Glycoside, and Antioxidant Phenolic Derivatives from *Tasmannia lanceolata*

**DOI:** 10.3390/molecules31193361

**Published:** 2026-09-22

**Authors:** Yuxuan Duan, Wei Li, Feifei Wang, Yihong Xu, Qingqiang Lin, Yulin Fang, Yongbiao Zheng, Rongbin Lin

**Affiliations:** 1 College of Life Sciences, Fujian Normal University, Fuzhou 350117, China; dyx_dione@126.com (Y.D.); l260215312@163.com (W.L.); wangfeifei1119@163.com (F.W.); 18859755102@163.com (Y.X.); qingqianglin@fjnu.edu.cn (Q.L.); 2 Institute of Edible Mushroom, Fujian Academy of Agricultural Sciences, Fuzhou 350014, China

**Keywords:** *Tasmannia lanceolata*, eudesmane sesquiterpene glycoside, phenolic compounds, antioxidant activity, DPPH assay, *α*-glucosidase inhibition, natural antioxidants

## Abstract

*Tasmannia lanceolata* (pepperberry) is a pungent plant rich in polyphenols with considerable potential as a natural antioxidant source. In this study, a phytochemical investigation of *T. lanceolata* extract was performed using column chromatography over RP-C_18_, silica gel, and Sephadex LH-20. Eight compounds were isolated, and their structures were elucidated by extensive spectroscopic analyses (1D and 2D NMR, HR-ESI-MS, UV, IR, CD, and ORD). Compound **1** was identified as a new eudesmane-type sesquiterpene glycoside, designated tasmannioside A, which exhibited *α*-glucosidase inhibitory activity with an inhibition rate of 21.63 ± 1.57% at 0.172 mM. The known compounds comprised one organic acid, shikimic acid (**2**); three phenolic acids, protocatechuic acid (**3**), methyl chlorogenate (**4**), and ethyl chlorogenate (**7**); one flavonoid, catechin-(5,6-bc)-4*α*/4*β*-(3,4-dihydroxyphenyl)-dihydro-2(3H)-pyranone (**5**); one phenylpropanoid glycoside, michehedyoside D (**6**); and one sesquiterpenoid, oplodiol (**8**). In the DPPH radical scavenging assay, compounds **2**–**7** demonstrated antioxidant activity, with the scavenging ratios of 0.04, 6.96, 4.57, 5.80, 1.97, and 3.15, respectively. Structure–activity relationship analysis revealed that the catechol moiety is critical for potent radical scavenging. These findings enrich the phytochemical diversity of *T. lanceolata* and provide preliminary in vitro evidence supporting further investigation of its phenolic constituents as natural antioxidants. However, in vivo efficacy, bioavailability, and long-term safety remain to be established.

## 1. Introduction

Oxidative stress, resulting from an imbalance between reactive oxygen species (ROS) production and antioxidant defense mechanisms, is implicated in the pathogenesis of numerous chronic diseases, including cardiovascular disorders, neurodegeneration, and cancer [1,2]. Although synthetic antioxidants such as butylated hydroxyanisole (BHA) and butylated hydroxytoluene (BHT) are widely used in food and pharmaceutical industries, growing concerns over their potential carcinogenicity and endocrine-disrupting effects have stimulated intensive research into safe and effective natural alternatives [3]. Plants, particularly those used as culinary spices and traditional medicines, represent a rich reservoir of polyphenolic antioxidants that can delay lipid peroxidation and scavenge free radicals [4].

*Tasmannia lanceolata* (R.Br.), commonly known as the Tasmanian pepperberry, is a pungent shrub endemic to southeastern Australia and Tasmania. Belonging to the family Winteraceae, it is phylogenetically distinct from the common black pepper (*Piper nigrum* L., Piperaceae) and is valued for its aromatic leaves and berries in both gastronomy and traditional medicine [5,6]. Previous phytochemical investigations have revealed that *T. lanceolata* is a prolific source of drimane-type sesquiterpenes—most notably polygodial, warburganal, and 1*β*-acetoxy-9-deoxy-isomuzigadial—which contribute to its characteristic pungency and exhibit broad-spectrum antimicrobial, gastroprotective, and transient receptor potential ankyrin 1 (TRPA1) channel-activating properties [7,8,9,10]. In addition to sesquiterpenes, the plant contains abundant polyphenols, including anthocyanins, flavonoids, and chlorogenic acid derivatives, which underlie its marked antioxidant capacity [11,12]. Comparative studies have ranked *T. lanceolata* among the highest in total phenolic content and oxygen radical absorbance capacity among native Australian plants, even surpassing conventional berries such as blueberry and blackberry [13].

Despite these promising findings, most existing studies on *T. lanceolata* have focused on crude extract profiling or total phenolic quantification, while systematic isolation and structural elucidation of individual antioxidant constituents remain scarce [10]. Consequently, the specific compounds responsible for the observed antioxidant activity and their quantitative structure–activity relationships (SARs) have not been fully clarified [14]. Furthermore, the *α*-D-glucoside glucohydrolase (*α*-glucosidase) inhibitory potential of *T. lanceolata* constituents, relevant to postprandial glycemic control, has been largely unexplored [4]. To address these gaps, the present study was designed to (i) isolate and purify bioactive constituents from a commercial *T. lanceolata* preparation using a combination of reversed-phase, normal-phase, and Sephadex LH-20 filtration chromatography; (ii) elucidate their structures by comprehensive spectroscopic methods (NMR, HR-ESI-MS, UV, IR, CD, and ORD); and (iii) evaluate their antioxidant capacity (DPPH and hydroxyl radical scavenging) and *α*-glucosidase inhibitory activity, with preliminary assessment of anti-inflammatory potential. These results are expected to expand the chemical diversity database of *T. lanceolata* and provide a preliminary basis for further investigation of its phenolic constituents as natural antioxidants.

## 2. Results and Discussion

### 2.1. Structure Elucidation

Eight compounds were isolated from the crude extract of *T. lanceolata* prepared as described in the Experimental section (Figures S33–S40). Their structures were unambiguously elucidated by extensive 1D and 2D Nuclear Magnetic Resonance (NMR) spectroscopy, high-resolution electrospray ionization mass spectrometry (HR-ESI-MS), and comparison with the literature data. Detailed spectroscopic data for all compounds are provided in the Supplementary Materials.

Compound **1** was obtained as a white powder with [α${]}_{D}^{25}$ +14.4 (*c* 0.10, MeOH) (Table S1) and the UV absorption (λmax) at 205 nm (log ε 3.61) (Figure S2). Its molecular formula was established as C_21_H_34_O_8_ by NMR data (Table 1 and Figures S3–S9) and HR-ESI-MS (positive-ion mode), which showed a sodiated molecular ion peak at *m/z* 437.2148 [M+Na]^+^ (calcd. for C_21_H_34_NaO_8_, 437.2151) (Figure S1), indicating five degrees of unsaturation. The IR spectrum displayed absorption bands at 3421 cm^−1^ (broad O–H stretching), 2929 and 2852 cm^−1^ (saturated C–H stretching), 1732 cm^−1^ (strong ester carbonyl C=O stretching), 1642 cm^−1^ (terminal alkene C=C stretching), and 1073 cm^−1^ (C–O–C stretching of the sugar ring) (Figure S11). The ^13^C NMR (Figure S3) and DEPT spectra (Figure S6) of **1** displayed 21 carbon signals, categorized as two methyl groups (δ_C_ 29.4, 14.6), one terminal methylene (δ_C_ 107.6), one ester carbonyl (δ_C_ 177.6), one hydroxymethyl (δ_C_ 59.0), one glucopyranosyl moiety (δ_C_ 95.6, 74.1, 78.5, 71.2, 78.8, 62.5), and ten additional aliphatic carbons comprising two quaternary carbons (δ_C_ 148.2, 40.7), three methines (δ_C_ 57.6, 59.1, 40.5), and five methylenes (δ_C_ 21.0, 39.1, 26.9, 39.5, 40.5). This carbon inventory, combined with the molecular formula, suggested an eudesmane-type sesquiterpene glycoside skeleton. Analysis of the ^1^H–^1^H COSY (Figure S5) spectrum established two independent spin systems: (i) H-1/H-2/H-3 for the A-ring and (ii) H-5/H-6/H-7/H-9 for the B-ring of the decalin system, together with the glucose spin system H-1′/H-2′/H-3′/H-4′/H-5′/H-6′. The large coupling constant of the anomeric proton H-1′ (δ_H_ 5.43, d, *J* = 8.1 Hz) indicated a *β*-configuration for the *D*-glucopyranosyl unit. Key HMBC correlations (Figure S7) from H-1′ (δ_H_ 5.43) and H-14 (δ_H_ 1.25) to C-15 (δ_C_ 177.6) demonstrated that the *β*-*D*-glucose moiety was attached via an ester glycosidic bond at C-4 through the C-15 carboxyl group. Correlations from H-13 (δ_H_ 4.89, 4.68) to C-7 (δ_C_ 39.5), C-8 (δ_C_ 148.2), and C-12 (δ_C_ 59.0) confirmed the location of the terminal double bond at C-8(13). Additionally, HMBC correlations from H-11 (δ_H_ 0.63) to C-1 (δ_C_ 40.5), C-5 (δ_C_ 57.6), and C-12 (δ_C_ 59.0) indicated that C-10 bears a methyl group (C-11) and C-9 bears a hydroxymethyl group (C-12). Comparison of the NMR spectroscopic data of **1** with those of koraienside A indicated that both compounds possess the same eudesmane sesquiterpene moiety (metaglyptin O), differing only in that koraienside A has a γ-lactone group attached at C-12 [δ_C_ 178.9, 80.3, 78.2, 42.9; δ_H_ 4.11 (2H, overlap), 2.59 (1H, dd, *J* = 17.2, 12.6 Hz), 2.37 (1H, dd, *J* = 17.2, 9.7 Hz)] [15], while **1** bears a hydroxyl group at the corresponding position, thereby establishing the core structure of **1**. The relative configuration was determined by analysis of the NOESY spectrum (Figure S9), which showed correlations between H-11 and H-12, placing these groups on the same face of the decalin system, and between H-5 and H-14, indicating that H3–14 and H-5 are co-facial. The absolute configuration of **1** was established as (15*S*, 5*R*, 9*S*, 10*R*) by comparison of its dextrorotatory optical rotation ([*α*${]}_{D}^{25}$ +14.4) with those of the structurally related eudesmane sesquiterpenes metaglyptin O ([*α*${]}_{D}^{20}$ +13) [16] and koraienside A ([*α*${]}_{D}^{25}$ +62), and was further supported by its CD spectrum displaying a positive Cotton effect at 215 nm (Δε +0.82) (Figure S10), similar to the ECD of koraienside A [(MeOH) λmax (Δε) 225 (+2.45) nm] [15].

A comprehensive SciFinder search confirmed that compound **1** is a new eudesmane-type sesquiterpene glycoside, for which the trivial name tasmannioside A is proposed (Figure 1). Notably, this eudesmane skeleton differs markedly from the drimane-type sesquiterpenes (e.g., polygodial) that dominate the literature on *T. lanceolata*, thereby expanding the structural diversity of sesquiterpenoids in this species [7,17].

In addition to the new compound **1**, seven known compounds were identified by direct comparison of their NMR and MS data with the literature data: shikimic acid (**2**, C_7_H_10_O_5_) [18], protocatechuic acid (**3**, C_7_H_6_O_4_) [19], methyl chlorogenate (**4**, C_17_H_20_O_9_) [20], catechin-(5,6-bc)-4*α*/4*β*-(3,4-dihydroxyphenyl)-dihydro-2(3H)-pyranone (**5**, C_24_H_20_O_9_) [21], michehedyoside D (**6**, C_16_H_22_O_8_) [22], ethyl chlorogenate (**7**, C_18_H_22_O_9_) [23], and oplodiol (**8**, C_15_H_26_O_2_) [24] (Figure 2 and Figures S12–S30). Among these, compounds **2**, **3**, **5**, **6**, and **8** are reported from *T. lanceolata* for the first time. Compounds **4** and **7** are chlorogenic acid derivatives; previous studies on *T. lanceolata* primarily reported total phenolics or the prototypical chlorogenic acid [11], so the isolation of these esterified derivatives complements the chemical inventory of caffeoylquinic acid constituents in this plant. Compound **5**, a catechin dimer flavonoid previously isolated only from *Eucommia ulmoides* leaves and recognized for its NF-*κ*B inhibitory activity [21], is of particular interest as a new chemotaxonomic marker for *T. lanceolata*. Compound **6**, originally obtained from *Michelia hedyosperma* and later from *Rosa soulieana* [22,25], further diversifies the phenylpropanoid glycoside profile of the Winteraceae family.

### 2.2. Antioxidant Activity

The antioxidant potential of the *T. lanceolata* crude extract and isolated compounds was evaluated by hydroxyl radical and DPPH radical scavenging assays. The crude extract (1%, *v/v*) exhibited a hydroxyl radical scavenging rate of 57.8 ± 1.0%, which was statistically significant compared with the blank control (*p* < 0.05) but lower than that of the positive control vitamin C (0.6 mg/mL, 78.6 ± 0.4%). Under the experimental conditions, the concentration of the crude extract required for 50% scavenging was approximately 1% (*v/v*), corresponding to an effective active compound concentration of approximately 200 ppm, whereas vitamin C required approximately 4000 ppm. This suggests that the crude extract possesses appreciable hydroxyl radical scavenging capacity at relatively low concentrations.

In the DPPH radical scavenging assay, the crude extract showed a concentration-dependent scavenging effect with an IC_50_ of 0.29% (*v/v*), compared with 0.08 mg/mL for the positive control vitamin E (VE). At dilutions below the original concentration, the extract exhibited a scavenging rate as high as 87.50%, indicating potent radical quenching ability even at low volume fractions [26].

To identify the specific antioxidant constituents, compounds **2**–**7** were subjected to TLC-DPPH bioautography and quantitative DPPH assays. In the TLC-DPPH assay, compounds **3**–**7** produced distinct white spots against a purple background, indicating strong antioxidant activity, whereas compound **2** (shikimic acid) showed only faint spots, suggestive of weak activity (Figure S31). Quantitative analysis revealed that when the DPPH radical scavenging rate reached 50%, the IC_50_ values of VC and compounds **2**–**7** were 0.402, 74.337, 0.401, 0.610, 0.481, 1.414, and 0.885 μg/mL, respectively (Figure S32 and Table S2). We further calculated the mass of DPPH scavenged per unit mass of antioxidant; the larger the value, the better the activity. The values of VC and compounds **2**–**7** were 6.94, 0.04, 6.96, 4.57, 5.80, 1.97, and 3.15, respectively. Compounds **3**–**7** exhibited strong antioxidant activity, with compound **3** showing slightly stronger activity than the positive control VC. In contrast, compound **2** only exhibited weak DPPH radical scavenging capacity.

The marked difference in antioxidant potency can be rationalized by SAR analysis. Compounds **3**–**7** all possess a catechol (1,2-dihydroxybenzene) moiety, whose phenolic hydroxyl groups can stabilize the resulting phenoxyl radical via intramolecular hydrogen bonding and resonance delocalization to ortho-quinone intermediates, thereby facilitating efficient radical scavenging [27,28]. Protocatechuic acid (**3**), with two free phenolic hydroxyl groups, showed an IC_50_ (0.401 μg/mL), which is stronger than that of VC in this experiment. Similarly, protocatechuic acid isolated from *Emilia sonchifolia* (25.57 μg/mL) also exhibits a stronger DPPH radical scavenging ability than VC [29]. Methyl chlorogenate (**4**) and ethyl chlorogenate (**7**), as caffeoylquinic acid esters, contain both catechol and additional hydroxyl groups on the quinic acid core; this multi-site hydrogen-donating capacity underlies their strong activity, consistent with previous reports on caffeoylquinic acid derivatives [30,31]. The flavonoid derivative **5** (catechin-(5,6-bc)-4*α*/4*β*-(3,4-dihydroxyphenyl)-dihydro-2(3H)-pyranone) and the phenylpropanoid glycoside **6** (michehedyoside D) also benefited from the catechol pharmacophore. Interestingly, compound **6** (IC_50_ = 1.414 μg/mL) exhibited stronger DPPH scavenging than a reported michehedyoside D analog from *Schisandra chinensis* (IC_50_ = 62.86 ± 2.31 μM) [32], which may reflect differences in assay protocols or synergistic matrix effects. In contrast, shikimic acid (**2**) lacks phenolic hydroxyl groups; its hydroxyl groups are alcoholic in nature, possessing higher O–H bond dissociation energies and an inability to form stable conjugated radical intermediates, thus explaining its weak activity [33]. Collectively, among the compounds tested, compounds **3**–**7** showed the strongest DPPH radical scavenging activity, suggesting that they may contribute to the antioxidant activity of *T. lanceolata*. However, their relative contribution requires further quantification.

### 2.3. α-Glucosidase Inhibitory and Anti-Inflammatory Activities of Compound ***1***

*α*-Glucosidase is a key intestinal enzyme responsible for the hydrolysis of dietary carbohydrates into absorbable monosaccharides; its inhibition represents a clinically validated strategy for managing postprandial hyperglycemia in type 2 diabetes mellitus [34]. The new eudesmane-type sesquiterpene glycoside **1** was therefore evaluated for *α*-glucosidase inhibitory activity. At a final concentration of 0.172 mM, compound **1** exhibited an *α*-glucosidase inhibition rate of 21.63 ± 1.57%, whereas the positive control acarbose (0.166 mM) showed 99.75 ± 0.13% inhibition (Table 2). Although the activity of **1** is modest compared with acarbose, it suggests that the eudesmane glycoside scaffold may serve as a lead structure for further optimization. However, the present data do not establish therapeutic potential, and additional validation is required.

Structure–activity relationship analysis provides a rationale for the observed weak inhibition. The C-9 hydroxymethyl group and the exocyclic methylene double bond in **1** may act as a hydrogen-bond donor and a hydrophobic interaction site, respectively, within the enzyme active site. However, two structural constraints likely attenuate potency: (i) esterification of the C-15 carboxyl group with glucose masks a carboxylate moiety that could otherwise engage in favorable electrostatic interactions with the catalytic residues of *α*-glucosidase [35]; and (ii) the bulky glucosyl substituent, while enhancing aqueous solubility, may reduce the hydrophobic interactions required for optimal enzyme binding [36]. These observations align with reports that glycosylation of terpenoid carboxylic acids often diminishes *α*-glucosidase inhibitory potency relative to their aglycones [37,38].

The anti-inflammatory potential of compound **1** was preliminarily assessed in lipopolysaccharide (LPS)-induced RAW264.7 murine macrophages. Cytotoxicity screening (CCK-8 assay) confirmed that compound **1** was non-cytotoxic to RAW264.7 cells at concentrations ranging from 1 to 200 μM. Under these non-cytotoxic conditions, however, compound **1** did not exhibit detectable inhibitory effects on LPS-induced nitric oxide (NO) production. This lack of activity can be rationalized by three structural factors. First, esterification of the C-15 carboxyl group with *β*-*D*-glucose precludes cyclization to form a Michael acceptor pharmacophore, a structural feature essential for covalent targeting of cysteine residues in NF-*κ*B and other inflammatory signaling proteins [39]. Second, the quaternary carbon substitution at C-4 introduces considerable steric hindrance that is unfavorable for target protein binding. Third, the isolated exocyclic methylene double bond lacks a conjugated unsaturated system, limiting antioxidant capacity and subsequent anti-inflammatory signaling [40]. It is noteworthy that while several eudesmane-type sesquiterpene glycosides have been reported to exert anti-inflammatory effects via NF-*κ*B pathway inhibition [41], structural similarity does not guarantee functional equivalence; the bioactivity of eudesmane glycosides is highly sensitive to the nature and position of substituents, as exemplified by andrographatoside and adenanthoside C, which share the same core skeleton yet show no significant antimicrobial or cytotoxic activity [42,43]. Further derivatization of the C-15 ester group or aglycone modification may be necessary to unlock the anti-inflammatory potential of this scaffold.

Nevertheless, these interpretations remain inferential, and further molecular studies are required to elucidate the specific mechanisms.

## 3. Materials and Methods

### 3.1. General Experimental Procedures

Nuclear Magnetic Resonance (NMR) spectra were recorded on a JEOL JNM-ECZ600R/S1 spectrometer (JEOL Ltd., Tokyo, Japan) operating at 600 MHz for ^1^H NMR and 151 MHz for ^13^C NMR, using tetramethylsilane (TMS) as an internal standard. Chemical shifts (δ) are expressed in parts per million (ppm) and coupling constants (*J*) in Hertz (Hz). HR-ESI-MS was performed on a SCIEX X500R QTOF system (SCIEX, Framingham, MA, USA) in positive-ion mode. Infrared (IR) spectra were obtained on a Bruker Tensor II FT-IR spectrometer (Bruker Corporation, Billerica, MA, USA) using KBr pellets. Ultraviolet (UV) spectra were measured on a Shimadzu UV-2600i spectrophotometer (Shimadzu Corporation, Kyoto, Japan). Optical rotations were determined with a Rudolph Research Analytical Autopol VI automatic polarimeter (Rudolph Research Analytical, Hackettstown, NJ, USA). Circular dichroism (CD) spectra were recorded on an Applied Photophysics Chirascan spectropolarimeter (Applied Photophysics Ltd., Leatherhead, UK). Column chromatography (CC) was performed on reversed-phase C_18_ silica gel (RP-C_18_, 40–63 μm, Merck Chemicals Shanghai Co., Ltd., Shanghai, China), normal-phase silica gel (200–300 mesh, Qingdao Haiyang Chemical Co., Ltd., Qingdao, China), and Sephadex LH-20 gel filtration medium (GE Healthcare/Cytiva, Marlborough, MA, USA). Thin-layer chromatography (TLC) was carried out on pre-coated silica gel GF_254_ plates (0.20–0.25 mm, Yantai Jiangyou Silicone Development Co., Ltd., Yantai, China), and spots were visualized under UV light (254 and 365 nm) and by spraying with 10% sulfuric acid in ethanol followed by heating. Medium-pressure preparative liquid chromatography (MPLC) was conducted on a QuikSep0050D-II system (H&E Co., Ltd., Beijing, China).

All solvents used for chromatography were of analytical grade and purchased from Sinopharm Chemical Reagent Co., Ltd. (Shanghai, China). Deuterated solvents for NMR measurements were methanol-d_4_ (PR-33736/051922, Qingdao Tenglong Weibo Technology Co., Ltd., Qingdao, China). Additionally, 1,1-diphenyl-2-picrylhydrazyl (DPPH) was the product of Aladdin Industrial Corporation, Shanghai, China (D1227007). *α*-Glucosidase was the product of Shanghai Yuanye Bio-Technology Co., Ltd., Shanghai, China (SLBV6748). Acarbose was the product of Bayer AG. Dexamethasone was the product of Shanghai Yuanye Bio-Technology Co., Ltd. (S17003). Dulbecco’s Modified Eagle Medium (DMEM) high-glucose basal medium (PM150210) and specialized culture medium for RAW264.7 cells (CM-0190) were the product of Procell Life Science Co., Ltd., Wuhan, China. CRD10 serum-free cell cryopreservation medium (CF0101) was the product of Cellregen Beijing Life Science and Technology Co., Ltd., Beijing, China. In the activity experiments, the following kits were used: hydroxyl radical scavenging activity assay kit (R3–344, Shanghai Yuanye Bio-Technology Co., Ltd.), CCK-8 assay kit (BS350B, BioSharp Biotechnology Co., Ltd., Beijing, China), and nitric oxide (NO) detection assay kit (S0021S, Beyotime Biotech Inc., Shanghai, China).

### 3.2. Plant Material

Fresh leaves of *T. lanceolata* were collected from Beyonte Planting Base, Sanming City (26.198° N latitude and 117.968° E longitude; elevation 589.08 m), Fujian Province, China (Figure S41). The botanical identity of the raw material was verified by morphological examination and comparison with herbarium specimens by Professor Yulin Fang (College of Life Sciences, Fujian Normal University). A voucher specimen (No. FNU-TB-2024-001) has been deposited at the Herbarium of the College of Life Sciences, Fujian Normal University, Fuzhou, China.

### 3.3. Extraction and Isolation

The *T. lanceolata* leaves (50 g), after defatting by n-hexane extraction, were subjected to maceration with 8 volumes (*v/w*) of ethanol–water (1:1, *v/v*) at 70 °C for 6 h. After filtration, the residue was rinsed with a small amount of ethanol, and the combined filtrates were concentrated to 150 g. The concentrate was partitioned with n-hexane–water (1:1, *v/v*) to remove residual non-polar components, predominantly dialdehyde-type compounds, that remained from the preceding hexane extraction. The aqueous phase was stored at 4 °C overnight; the precipitated solids were removed by filtration the next day. The resulting filtrate was passed through an NDA-88 macroporous adsorption resin column, with the adsorption process repeated 1–2 times to ensure sufficient binding. The resin was then washed with deionized water until no impurities were detected in the effluent, and subsequently dried by pressing to remove excess water. Finally, the adsorbed constituents were eluted with ethanol to obtain the *T. lanceolata* solution.

A 500 mL aliquot of the *T. lanceolata* solution was concentrated under reduced pressure at 45 °C using a Büchi R-300 rotary evaporator (Büchi Labortechnik AG, Flawil, Switzerland) to yield 16.8 g of a brown, pasty crude extract. A portion of the crude extract (15.0 g) was subjected to reversed-phase column chromatography (RP-C_18_, 150 g) eluted with a stepwise gradient of methanol (MeOH) in water (H_2_O): 100% H_2_O, 30% MeOH, 50% MeOH, and 70% MeOH. This separation afforded six primary fractions: Fr.1 (95.4 mg, 100% H_2_O), Fr.2 (80.7 mg, 100% H_2_O), Fr.3 (3.7014 g, 30% MeOH), Fr.4 (510.2 mg, 30% MeOH), Fr.5 (221.7 mg, 50% MeOH), and Fr.6 (448.6 mg, 70% MeOH).

Fr.5 (221.7 mg) was chromatographed over a Sephadex LH-20 gel column (120 g) eluted with pure MeOH to afford two subfractions: Fr.5.1 (50.8 mg) and Fr.5.2 (26.1 mg). Fr.5.1 was further purified by reversed-phase column chromatography (MeOH–H_2_O, 40:60, *v/v*) to give Fr.5.1.1 (22.0 mg), which was subsequently subjected to normal-phase silica gel column chromatography (CH_2_Cl_2_–MeOH, 50:1, *v/v*) to yield compound **1** (5.2 mg) as a white powder.

Fr.1 (95.4 mg) was chromatographed on Sephadex LH-20 (120 g, MeOH) to give Fr.1.1 (12.1 mg), which was purified by normal-phase silica gel column chromatography (CH_2_Cl_2_–MeOH, 40:1, *v/v*) to afford compound **2** (4.0 mg).

Fr.2 (80.7 mg) was separated on Sephadex LH-20 (120 g, MeOH) to afford Fr.2.1 (17.1 mg). Normal-phase silica gel column chromatography (CH_2_Cl_2_–MeOH, 200:1, *v/v*) of Fr.2.1 yielded compound **3** (5.6 mg).

A portion of Fr.3 (533.4 mg) was applied to a Sephadex LH-20 column (120 g, MeOH) to give Fr.3.1 (70.4 mg). Fr.3.1 (35.0 mg) was subjected to normal-phase silica gel column chromatography (CH_2_Cl_2_–MeOH, 40:1, *v/v*) to yield compound **4** (12.7 mg).

Fr.4 (510.2 mg) was fractionated by Sephadex LH-20 gel column chromatography (120 g, MeOH) to afford Fr.4.1 (40.1 mg) and Fr.4.2 (14.3 mg). Fr.4.2 was purified by normal-phase silica gel column chromatography (CH_2_Cl_2_–MeOH, 40:1, *v/v*) to obtain compound **5** (5.2 mg). Fr.4.1 was further separated by reversed-phase column chromatography (MeOH–H_2_O, 30:70, *v/v*) to yield Fr.4.1.1 (14.5 mg), which was subsequently chromatographed on normal-phase silica gel (CH_2_Cl_2_–MeOH, 20:1, *v/v*) to afford compound **6** (2.9 mg).

Fr.5.2 (26.1 mg) was subjected to normal-phase silica gel column chromatography (CH_2_Cl_2_–MeOH, 60:1, *v/v*) to yield compound **7** (5.0 mg).

Fr.6 (448.6 mg) was loaded onto a Sephadex LH-20 column (120 g, MeOH) to afford Fr.6.1 (101.1 mg). This fraction was purified by reversed-phase column chromatography (MeOH–H_2_O, 60:40, *v/v*) to give Fr.6.1.1 (14.4 mg), which was further chromatographed on normal-phase silica gel eluted with petroleum ether–ethyl acetate (30:1, *v/v*) to yield compound **8** (1.6 mg).

Tasmannioside A (**1**): C_21_H_34_O_8_, white powder, [α${]}_{D}^{25}$+14.4 (c 0.100, MeOH); UV (MeOH) *λ*max (log *ε*) 205 (3.61) nm; CD (MeOH) *λ*_max_ (∆ε) 215 (+0.82) nm; IR (KBr) *ν*_max_3421, 2929, 2852, 1732, 1642, 1384, 1073 cm^−1^; ^1^H (600 MHz) and ^13^C (151 MHz) NMR data are listed in Table 1; HR-ESI-MS (positive-ion mode) m/z [M+Na]^+^ 437.2148 (calcd. for C_21_H_34_NaO_8_, 437.2151).

Shikimic acid (**2**): C_7_H_10_O_5_, pale yellow powder. ^1^H NMR (600 MHz, CD_3_OD)δ: 6.79 (1H, dd, *J* = 3.8, 1.9 Hz, H-2), 4.37 (1H, d, *J* = 4.0 Hz, H-3), 3.99 (1H, q, *J* = 5.9 Hz, H-5), 3.67 (1H, dd, *J* = 7.4, 4.2 Hz, H-4), 2.70 (1H, dd, *J* = 18.3, 4.8 Hz, H-6), 2.19 (1H, dd, *J* = 18.2, 5.6 Hz, H-6). ^13^C NMR (151 MHz, CD_3_OD)δ: 130.9 (C-1), 138.7 (C-2), 67.4 (C-3), 68.4 (C-4), 72.8 (C-5), 31.7 (C-6), 170.2 (C=O).

Protocatechuic acid (**3**): C_7_H_6_O_4_, white powder. ^1^H NMR (600 MHz, CD_3_OD)δ: 7.43 (1H, dd, *J* = 9.7, 2.0 Hz, H-2), 7.43 (1H, dd, *J* = 9.7, 2.0 Hz, H-6), 6.80 (1H, dd, *J* = 8.2, 1.8 Hz, H-5). ^13^C NMR (151 MHz, CD_3_OD)δ: 123.2 (C-1), 115.8 (C-2), 146.1 (C-3), 151.5 (C-4), 117.7 (C-5), 123.9 (C-6), 170.3 (C=O).

Methyl chlorogenate (**4**): C_17_H_20_O_9_, pale yellow powder. HR-ESI-MS: *m/z* 369.1175 [M+H]^+^ (calcd. for C_17_H_21_O_9_, 369.1186), 391.0990 [M+Na]^+^ (calcd. for C_17_H_20_NaO_9_, 391.1005). ^1^H NMR (600 MHz, CD_3_OD)δ: 7.49 (1H, d, *J* = 15.9 Hz, CH=CHCO), 7.00 (1H, d, *J* = 2.0 Hz, H-2′), 6.91 (1H, dd, *J* = 8.2, 2.1 Hz, H-6′), 6.74 (1H, d, *J* = 8.2 Hz, H-5′), 6.18 (1H, d, *J* = 15.9 Hz, CH=CHCO), 5.23 (1H, td, *J* = 7.4, 4.4 Hz, H-3), 4.10 (1H, dt, *J* = 6.9, 3.4 Hz, H-5), 3.69 (1H, dd, *J* = 7.4, 3.1 Hz, H-4), 3.65 (3H, s, MeO), 2.17 (1H, m, H-6), 2.15 (1H, m, H-2), 2.11 (1H, dd, *J* = 13.6, 7.9 Hz, H-6), 1.97 (1H, dd, *J* = 13.6, 7.0 Hz, H-2). ^13^C NMR (151 MHz, CD_3_OD)δ: 75.8 (C-1), 37.7 (C-2), 72.1 (C-3), 72.5 (C-4), 70.3 (C-5), 38.0 (C-6), 127.6 (C-1′), 115.1 (C-2′), 146.9 (C-3′), 149.7 (C-4′), 116.5 (C-5′), 123.0 (C-6′), 175.4 (COOMe), 168.3 (CH=CHCO), 147.2 (CH=CHCO), 115.0 (CH=CHCO), 53.0 (COOMe).

Catechin-(5,6-bc)-4*α*/4*β*-(3,4-dihydroxyphenyl)-dihydro-2(3H)-pyranone (**5**): C_24_H_20_O_9_, white powder. HR-ESI-MS: *m/z* 453.1179 [M+H]^+^ (calcd. for C_24_H_21_O_9_, 453.1186). ^1^H NMR (600 MHz, CD3OD)δ: 6.84 (1H, d, *J* = 2.1 Hz, H-2′), 6.77 (1H, d, *J* = 8.2 Hz, H-5′), 6.72 (1H, dd, *J* = 8.3, 2.1 Hz, H-6′), 6.66 (1H, d, *J* = 8.2 Hz, H-5″), 6.55 (1H, m, H-2″), 6.47 (1H, dd, *J* = 8.8, 2.4 Hz, H-6″), 6.22 (1H, m, H-8), 4.70 (1H, m, H-2), 4.45 (1H, d, *J* = 6.6 Hz, H-β), 4.08 (1H, m, H-3), 3.03 (1H, m, H-*α*), 2.96 (1H, m, H-4β), 2.88 (1H, m, H-*α*), 2.66 (1H, m, H-4*α*). ^13^C NMR (151 MHz, CD_3_OD)δ: 82.9 (C-2), 68.1 (C-3), 27.7 (C-4), 101.5 (C-4a), 151.9 (C-5), 107.2 (C-6), 154.8 (C-7), 99.8 (C-8), 155.8 (C-8a), 131.9 (C-1′), 115.1 (C-2′), 145.2 (C-3′), 146.3 (C-4′,3″,4″), 116.2 (C-5′), 119.8 (C-6′), 38.4 (C-*α*), 35.2 (C-β), 134.9 (C-1″), 115.0 (C-2″), 116.5 (C-5″), 119.3 (C-6″), 170.3 (-COO-).

Michehedyoside D (**6**): C_16_H_22_O_8_, white powder. HR-ESI-MS: *m/z* 365.1198 [M+Na]^+^ (calcd. for C_16_H_22_NaO_8_, 365.1212). ^1^H NMR (600 MHz, CD_3_OD)δ: 6.70 (1H, d, *J* = 1.9 Hz, H-2), 6.53 (1H, d, *J* = 1.9 Hz, H-5), 5.95 (1H, m, H-8), 5.06 (1H, m, H-9), 5.02 (1H, dt, *J* = 10.1, 1.8 Hz, H-9), 4.75 (1H, d, *J* = 7.4 Hz, H-1′), 3.88 (1H, dd, *J* = 12.0, 1.8 Hz, H-6′), 3.82 (3H, s, H-OCH_3_), 3.71 (1H, dd, *J* = 12.1, 4.5 Hz, H-6′), 3.49 (1H, dd, *J* = 9.1, 7.4 Hz, H-6′), 3.45 (1H, m, H-5′), 3.40 (1H, m, H-3′), 3.39 (1H, m, H-2′), 3.28 (1H, d, *J* = 6.8 Hz, H-7), 3.28 (1H, d, *J* = 6.8 Hz, H-4′). ^13^C NMR (151 MHz, CD_3_OD)δ: 135.8 (C-1), 108.5 (C-2), 149.6 (C-3), 132.5 (C-4), 146.9 (C-5), 111.7 (C-6), 41.02 (C-7), 139.1 (C-8), 115.8 (C-9), 104.3 (C-1′), 74.9 (C-2′), 77.6 (C-3′), 71.3 (C-4′), 78.3 (C-5′), 62.4 (C-6′), 56.7 (C-OCH_3_).

Ethyl chlorogenate (**7**): C_18_H_22_O_9_, pale yellow powder. HR-ESI-MS: *m/z* 383.1337 [M+H]^+^ (calcd. for C_18_H_23_O_9_, 383.1342), 405.115 0 [M+Na]^+^ (calcd. for C_18_H_22_NaO_9_, 405.116 2). ^1^H NMR (600 MHz, CD_3_OD)δ: 7.53 (1H, dd, *J* = 15.8, 1.5 Hz, H-7′), 7.04 (1H, t, *J* = 1.8 Hz, H-2′), 6.95 (1H, dt, *J* = 8.3, 1.8 Hz, H-6′), 6.78 (1H, dd, *J* = 8.2, 1.5 Hz, H-5′), 6.22 (1H, dd, *J* = 15.9, 1.6 Hz, H-8′), 5.28 (1H, q, *J* = 6.8 Hz, H-3), 4.13 (2H, m, -CH_2_CH_3_), 4.11 (1H, m, H-5), 3.73 (1H, m, H-4), 2.19 (2H, m, H-2), 2.19 (1H, m, H-6), 2.00 (1H, dd, *J* = 13.7, 6.9 Hz, H-6), 1.24 (3H, td, *J* = 7.1, 1.5 Hz, -CH_2_CH_3_). ^13^C NMR (151MHz, CD_3_OD)δ: 75.7 (C-1), 38.0 (C-2), 72.2 (C-3), 72.6 (C-4), 70.5 (C-5), 37.5 (C-6), 175.0 (C-7), 127.6 (C-1′), 115.1 (C-2′), 146.9 (C-3′), 149.7 (C-4′), 116.5 (C-5′), 123.0 (C-6′), 147.2 (C-7′), 115.0 (C-8′), 168.3 (C-9′), 62.5 (-CH_2_CH_3_), 14.3 (-CH_2_CH_3_).

Oplodiol (**8**): C_15_H_26_O_2_, white powder. HR-ESI-MS: *m/z* 221.1900 [M+H-H_2_O]^+^ (calcd. for C_15_H_25_O^+^, 221.1905). ^1^H NMR (600 MHz, CD_3_OD)δ: 5.32(1H, d, *J* = 5.7 Hz, H-8), 3.22 (1H, dd, *J* = 12.1, 3.7 Hz, H-1), 1.14 (3H, s, H-14), 1.04 (3H, s, H-12), 1.03 (3H, d, *J* = 3.1 Hz, H-13), 0.95 (3H, s, H-15). ^13^C NMR (151 MHz, CD_3_OD)δ: 80.8 (C-1), 42.0 (C-2), 40.4 (C-3), 71.5 (C-4), 47.9 (C-5), 27.6 (C-6), 143.4 (C-7), 117.2 (C-8), 24.1 (C-9), 39.0 (C-10), 36.4 (C-11), 22.2 (C-12), 21.7 (C-13), 29.9 (C-14), 12.2 (C-15).

### 3.4. Antioxidant Activity Assays

#### 3.4.1. Hydroxyl Radical Scavenging Assay

The assay was carried out according to the kit manufacturer’s instruction. Crude extract was diluted to 1% (*v/v*) with distilled water, and VC (0.6 mg/mL) served as positive control. Working solution (1,10-phenanthroline: PBS: Fe^2+^ = 2:1:2, *v/v*/*v*) was mixed with H_2_O_2_ and sample solution, incubated at 37 °C for 60 min and centrifuged, and absorbance was measured at 536 nm. Hydroxyl radical scavenging rate (%) = [(A_sample_ − A_control_)/(A_blank_ − A_control_)] × 100%. All assays were performed in three independent experiments (*n* = 3). Data of the hydroxyl radical scavenging rate are expressed as mean ± standard deviation (SD). Statistical analysis was conducted via one-way analysis of variance (one-way ANOVA) followed by Tukey’s post hoc test using SPSS 26.0; *p* < 0.05 was defined as statistically significant.

#### 3.4.2. DPPH Radical Scavenging Assay

Qualitative TLC-DPPH Bioautography*:* Each pure compound was dissolved in methanol to 1 mg/mL, and 10 μL sample solution and VC standard were spotted on TLC plates. After development, plates were sprayed with 0.4 mg/mL DPPH ethanol solution, dried, and heated at 40 °C for 30 min. White spots against purple background represented antioxidant active components.

Quantitative DPPH Assay: In total, 35.7 μg/mL DPPH ethanol solution was prepared. VC and compounds **3**–**7** were serially two-fold diluted from initial concentration 0.0625 mg/mL; compound **2** was diluted from 4 mg/mL. In total, 14 μL sample solution was mixed with 94 μL ethanol and 20 μL DPPH solution and incubated in dark at room temperature for 30 min, with absorbance recorded at 519 nm. DPPH radical scavenging rate (%) = [1 − (A_i_ − A_j_)/A_c_] × 100%, where A_c_ is the absorbance of the blank control; A_i_ is the absorbance of the sample group; and A_j_ is the absorbance of the solvent blank. All assays were performed in three independent experiments (*n* = 3). Data of the DPPH radical scavenging rate are expressed as mean ± SD. Statistical analysis was conducted via one-way ANOVA followed by Tukey’s post hoc test using SPSS 26.0; *p* < 0.05 was defined as statistically significant. IC_50_ values were calculated by nonlinear regression of concentration-scavenging rate curves using GraphPad Prism 9.0. Then, upon reaching 50% DPPH scavenging, the mass of DPPH scavenged per unit mass of antioxidant was calculated and defined as the scavenging ratio [44]. A higher scavenging ratio indicates stronger antioxidant activity.

### 3.5. α-Glucosidase Inhibitory Activity Assay

Compound **1** was dissolved in methanol to 1 mg/mL, with acarbose (1.5 mg/mL) as positive control. The reaction system contained PBS buffer (pH 6.8), sample solution, PNPG substrate, and *α*-glucosidase solution. Incubation was performed at 37 °C for 30 min and terminated with 0.1 mol/L Na_2_CO_3_, with absorbance detected at 405 nm. *α*-Glucosidase inhibition rate (%) = [1 − (A_1_ − A_2_)/(A_3_ − A_4_)] × 100%. A_1_ = sample absorbance; A_2_ = sample blank; A_3_ = negative control; and A_4_ = blank control. All groups *n* = 3.

### 3.6. Cytotoxicity and Anti-Inflammatory Assay on RAW264.7 Macrophages

#### 3.6.1. CCK-8 Cytotoxicity Test

RAW264.7 cells were seeded into 96-well plates and treated with gradient concentrations of compound **1** (1, 10, 50, 100, 200 μM) for 24 h. CCK-8 reagent was added and incubated for 60 min, and absorbance was measured at 450 nm to calculate cell viability.

#### 3.6.2. LPS-Induced NO Release Inhibition Assay

Cells were pretreated with compound **1** for 4 h, then stimulated by LPS (final concentration 300 ng/mL) for 24 h. Cell supernatant was collected and mixed with Griess reagent, and absorbance was detected at 540 nm to quantify NO concentration from standard curve. Dexamethasone was used as positive control, with five biological replicates per group (*n* = 5).

All biological experiments were performed in triplicate (*n* = 3), except for the LPS-induced NO release inhibition assay, which was performed with five biological replicates (*n* = 5). Data are expressed as mean ± SD. Statistical analysis was conducted via one-way ANOVA followed by Tukey’s post hoc test using SPSS 26.0; *p* < 0.05 was defined as statistically significant.

## 4. Conclusions

Eight isolated compounds, including one new eudesmane-type sesquiterpene glycoside tasmannioside A (**1**) and seven known compounds, were isolated and structurally identified from *T. lanceolata* extract. Five compounds were reported from this plant for the first time, enriching its phytochemical profile and sesquiterpenoid structural diversity. In vitro activity screening revealed that phenolic derivatives protocatechuic acid (**3**), methyl chlorogenate (**4**), ethyl chlorogenate (**7**), catechin-(5,6-bc)-4*α*/4*β*-(3,4-dihydroxyphenyl)-dihydro-2(3H)-pyranone (**5**), and michehedyoside D (**6**) exhibited strong DPPH radical scavenging activity comparable to vitamin C. These results indicate that compounds **3**–**7** are notable antioxidant constituents among the isolated compounds and suggest that they may contribute to the antioxidant capacity of the crude extract. The novel sesquiterpene glycoside tasmannioside A (**1**) displayed weak *α*-glucosidase inhibitory activity. In LPS-stimulated RAW264.7 macrophages, tasmannioside A (**1**) did not detectably inhibit NO production at non-cytotoxic concentrations; however, since only NO was measured, potential effects of tasmannioside A (**1**) on other inflammatory mediators or signaling pathways remain unconfirmed. The weak *α*-glucosidase inhibition and the absence of detectable NO inhibition were tentatively interpreted through SAR analysis based on structural features and the literature precedents. These SAR interpretations remain inferential and were not validated by molecular docking, molecular dynamics simulations, binding free energy calculations, or enzyme kinetic studies. Although this scaffold may merit further investigation, its therapeutic potential remains to be validated. This study systematically clarifies the monomeric chemical constituents and their corresponding bioactivities of *T. lanceolata*, and provides preliminary in vitro evidence that its phenolic constituents are promising candidates for further investigation as natural antioxidants. Nevertheless, several limitations should be acknowledged. Future studies should quantify the abundance of compounds **3**–**7** in the plant material and active fractions and elucidate the molecular mechanisms underlying their antioxidant effects. For the new compound **1**, further molecular experiments, such as molecular docking, molecular dynamics simulations, and enzyme kinetic analyses, are warranted to provide stronger support for its proposed structure–activity relationships. In addition, a more comprehensive evaluation of its biological activities, encompassing a broader panel of in vitro and in vivo assays, is needed to better define its pharmacological potential.

## Figures and Tables

**Figure 1:**
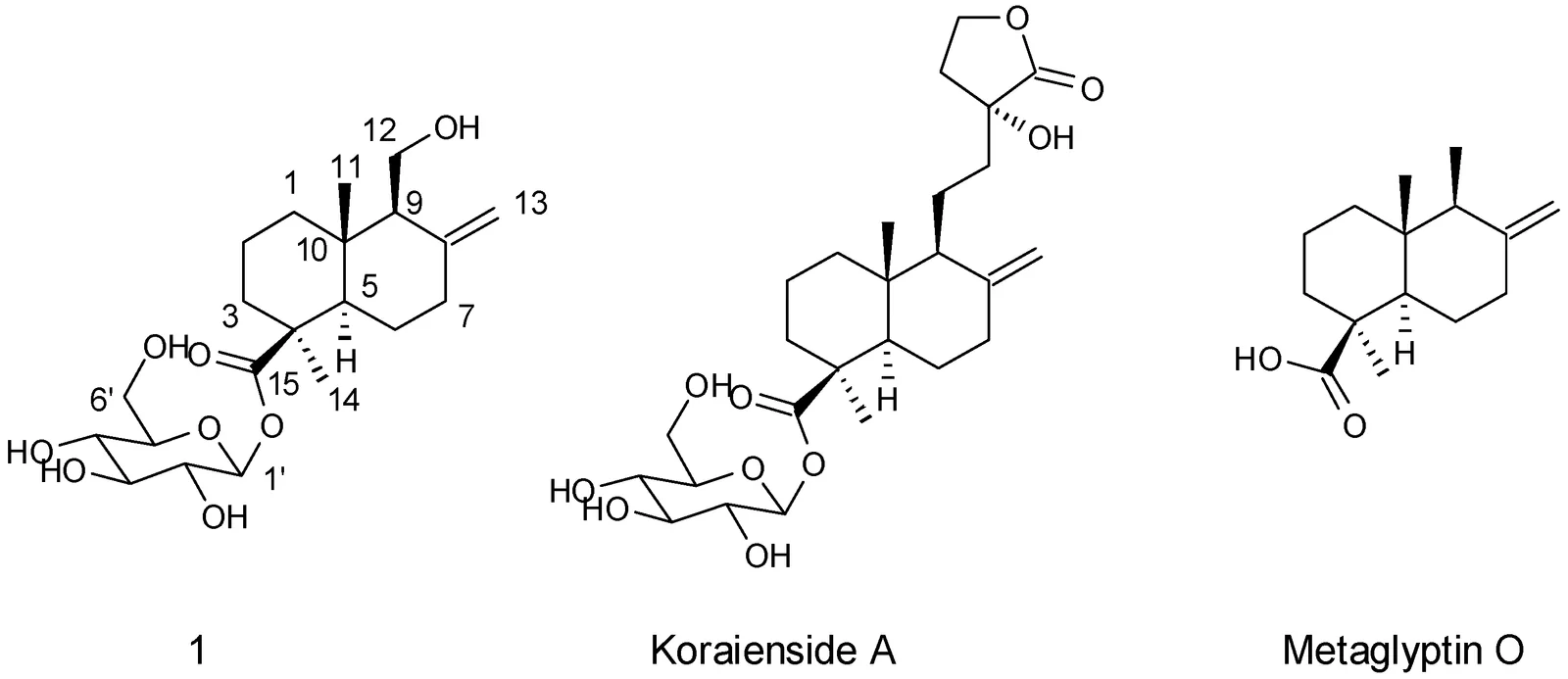
Chemical structures of tasmannioside A (**1**), koraienside A, and metaglyptin O.

**Figure 2:**
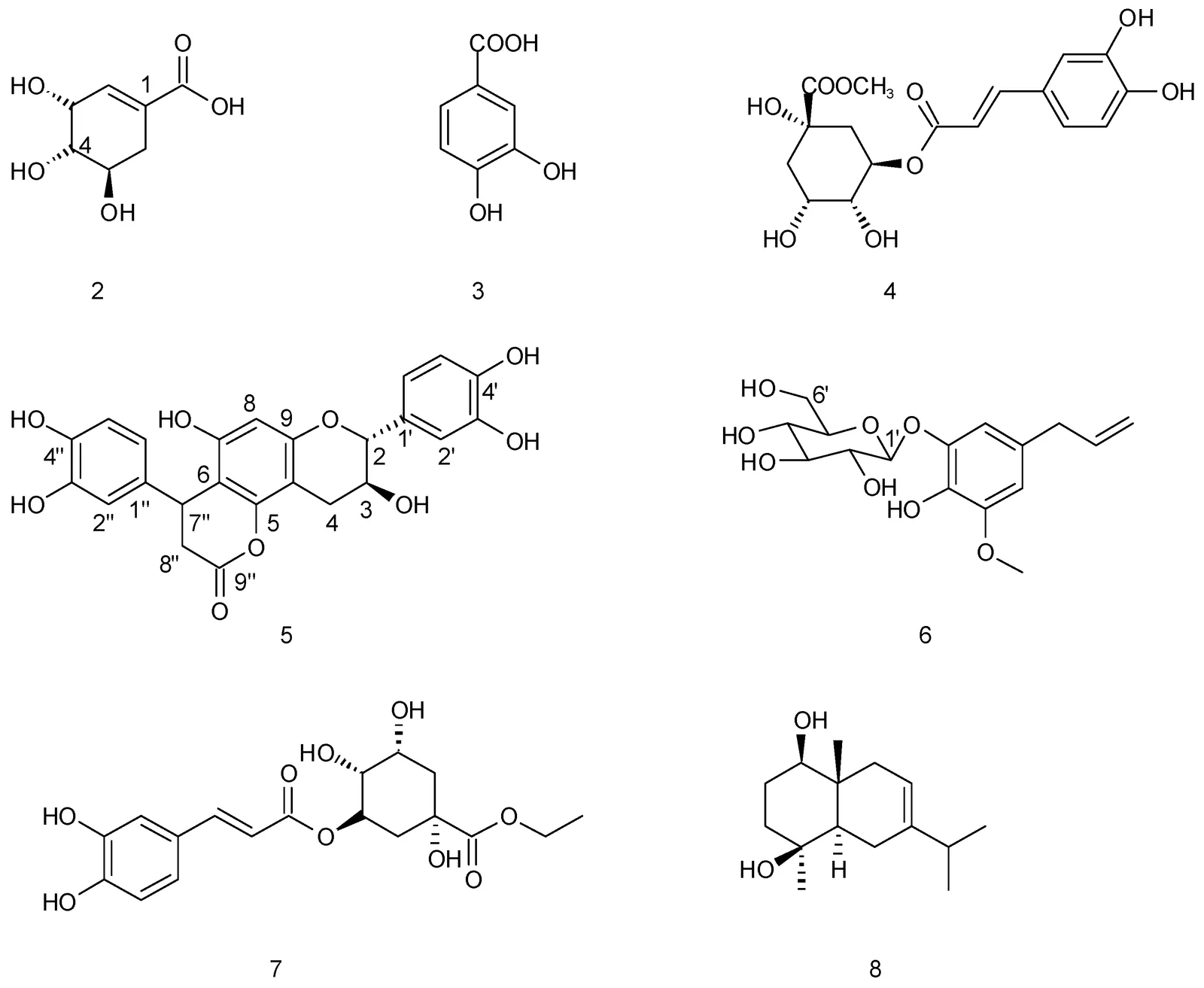
Chemical structures of compounds **2**–**8.**

**Table 1:** NMR data of compound **1** (CD_3_O).

Position	^1^H (600 MHz)	^13^C (151 MHz)	HMBC	COSY
1	1.84(m, 1H); 1.31 (dd, *J* = 13.6, 4.1 Hz, 1H)	40.5t		H-11, H-14, H-2, H-3
2	1.95 (m, 1H); 1.51 (m, 1H)	21.0t	C-8	H-11, H-5, H-2, H-3, H-1
3	2.21 (dt, *J* = 13.0, 3.4 Hz, 1H); 1.13 (td, *J* = 13.5, 3.9 Hz, 1H)	39.1t	C-5; C-15	H-14, H-2
4		45.6s	C-8	
5	1.42 (dd, *J* = 12.1, 2.9 Hz, 1H)	57.6d		H-3, H-14, H-1
6	2.02 (td, *J* = 9.9, 8.5, 4.5 Hz, 1H); 1.90 (m, 1H)	26.9t	C-1, C-8	H-14, H-5
7	2.42 (m, 1H); 1.95 (m, 1H)	39.5t	C-5, C-8; C-8	H-11, H-5, H-2
8		148.2s		
9	1.88 (m, 1H)	59.1d	C-15	H-5
10		40.7s		
11	0.63 (s, 3H)	14.6q	C-1, C-5, C-12	
12	3.82 (m, 1H); 3.69 (m, 1H)	59.0t	C-8; C-8, C-9	
13	4.89 (m, 1H); 4.68 (s, 1H)	107.6t	C-7, C-12; C-7, C-12, C-8	H-7, H-6′
14	1.25 (s, 3H)	29.4q	C-3, C-4, C-5, C-15	H-11, H-3
15		177.6s		
1′	5.43 (d, *J* = 8.1 Hz, 1H)	95.6d	C-15	H-5′
2′	3.33 (m, 1H)	74.1d	C-3′	
3′	3.39 (m, 1H)	78.5d	C-4′	
4′	3.36 (m, 1H)	71.2d	C-5′	
5′	3.34 (m, 1H)	78.8d	C-1′	
6′	3.80 (m, 1H); 3.67 (m, 1H)	62.5t	C-8; C-8, C-9	H-11, H-1, H-5′, H-12

**Table 2:** *α*-Glucosidase inhibitory of compound **1.**

Compound	Concentration (mM)	*α*-Glucosidase Inhibition Rate (%)
Tasmannioside A (**1**)	0.172	21.63 ± 1.57
Acarbose	0.166	99.75 ± 0.13

Note: Values are expressed as mean ± SD (*n* = 3).

## Data Availability

The data presented in this study are available in the Supplementary Materials and in the repository database.

## Supplementary Materials

The following supporting information can be downloaded at: https://www.mdpi.com/article/10.3390/molecules31193361/s1, Figure S1: The HR-ESI-MS (MeOH) spectrum of **1**; Figure S2: UV (MeOH) spectrum of **1**; Figure S3: The ^13^C NMR spectrum of **1** in CD_3_OD (151 MHz); Figure S4: The ^1^H NMR spectrum of **1** in CD_3_OD (600 MHz); Figure S5: The ^1^H-^1^H COSY spectrum of **1** in CD_3_OD; Figure S6: The DEPT spectrum of **1** in CD_3_OD; Figure S7: The HMBC spectrum of **1** in CD_3_OD; Figure S8: The HSQC spectrum of **1** in CD_3_OD; Figure S9: The NOESY spectrum of **1** in CD_3_OD; Figure S10: The CD spectrum of **1**; Figure S11: The IR spectrum of **1**; Figure S12: The ^13^C NMR spectrum of **2** in CD_3_OD (151 MHz); Figure S13: The ^1^H NMR spectrum of **2** in CD_3_OD (600 MHz); Figure S14: The ^13^C NMR spectrum of **3** in CD_3_OD (151 MHz); Figure S15: The ^1^H NMR spectrum of **3** in CD_3_OD (600 MHz); Figure S16: The HR-ESI-MS (MeOH) spectrum of **4**; Figure S17: The ^13^C NMR spectrum of **4** in CD_3_OD (151 MHz); Figure S18: The ^1^H NMR spectrum of **4** in CD_3_OD (600 MHz); Figure S19: The HR-ESI-MS (MeOH) spectrum of **5**; Figure S20: The ^13^C NMR spectrum of **5** in CD_3_OD (151 MHz); Figure S21: The ^1^H NMR spectrum of **5** in CD_3_OD (600 MHz); Figure S22: The HR-ESI-MS (MeOH) spectrum of **6**; Figure S23: The ^13^C NMR spectrum of **6** in CD_3_OD (151 MHz); Figure S24: The ^1^H NMR spectrum of **6** in CD_3_OD (600 MHz); Figure S25: The HR-ESI-MS (MeOH) spectrum of **7**; Figure S26: The ^13^C NMR spectrum of **7** in CD_3_OD (151 MHz); Figure S27: The ^1^H NMR spectrum of **7** in CD_3_OD (600 MHz); Figure S28: The HR-ESI-MS (MeOH) spectrum of **8**; Figure S29: The ^13^C NMR spectrum of **8** in CD_3_OD (151 MHz); Figure S30: The ^1^H NMR spectrum of **8** in CD_3_OD (600 MHz); Figure S31: Antioxidant activity of compounds **2**–**7**; Figure S32: The DPPH radical scavenging activity of compounds **3**-**7**. Data are presented as mean ± SD (*n* = 3); Figure S33: TLC analysis of compound **1** (CHCl_3_:MeOH=7:1) (Note: From left to right, the colors are UV 254 nm, UV 365 nm, iodine, concentrated sulfuric acid, and potassium bismuth iodide); Figure S34: TLC analysis of compound **2** (CHCl_3_:MeOH=4:1); Figure S35: TLC analysis of compound **3** (CHCl_3_:MeOH=4:1); Figure S36: TLC analysis of compound **4** (CHCl_3_:MeOH=5:1); Figure S37: TLC analysis of compound **5** (CHCl_3_:MeOH=3:1); Figure S38: TLC analysis of compound **6** (CHCl_3_:MeOH=3:1); Figure S39: TLC analysis of compound **7** (CHCl_3_:MeOH=7:1); Figure S40: TLC analysis of compound **8** (CHCl_3_:MeOH=10:1); Figure S41: Morphology of the *T. lanceolata* used as plant material in this study; Table S1: ORD data of **1** (CD_3_OD); Table S2: DPPH radical scavenging activity of compounds **2**–**7** at different concentrations.
