# Supplementary material for: Tasmannioside A, a New Eudesmane Sesquiterpene Glycoside, and Antioxidant Phenolic Derivatives from Tasmannia lanceolata
Source: Molecules. 2026 Sep 22;31(19):3361. doi: 10.3390/molecules31193361 (PMC13665859; doi:10.3390/molecules31193361)
Supplement: Supplementary file 1 [file molecules-31-03361-s001.zip › molecules-4584481-supplementary.pdf]

Electronic Supplementary Information

Tasmannioside A, a New Eudesmane Sesquiterpene Glycoside, and Antioxidant Phenolic Derivatives from *Tasmannia lanceolata*

Yuxuan Duan [1], Wei Li [1], Feifei Wang [1], Yihong Xu [1], Qingqiang Lin [1], Yulin Fang [1,*], Yongbiao Zheng [1,*] and Rongbin Lin [2,*]

[1] College of Life Sciences, Fujian Normal University, Fuzhou 350117, China; dyx_dione@126.com (Y.D.); l260215312@163.com (W.L.); wangfeifei1119@163.com (F.W.); 18859755102@163.com (Y.X.); qingqianglin@fjnu.edu.cn (Q.L.)

[2] Institute of Edible Mushroom, Fujian Academy of Agricultural Sciences, Fuzhou 350014, China

* Correspondence: fangyulin@fjnu.edu.cn (Y.F.); yongbiaozheng@fjnu.edu.cn (Y.Z.); 13960817200@sina.cn (R.L.)

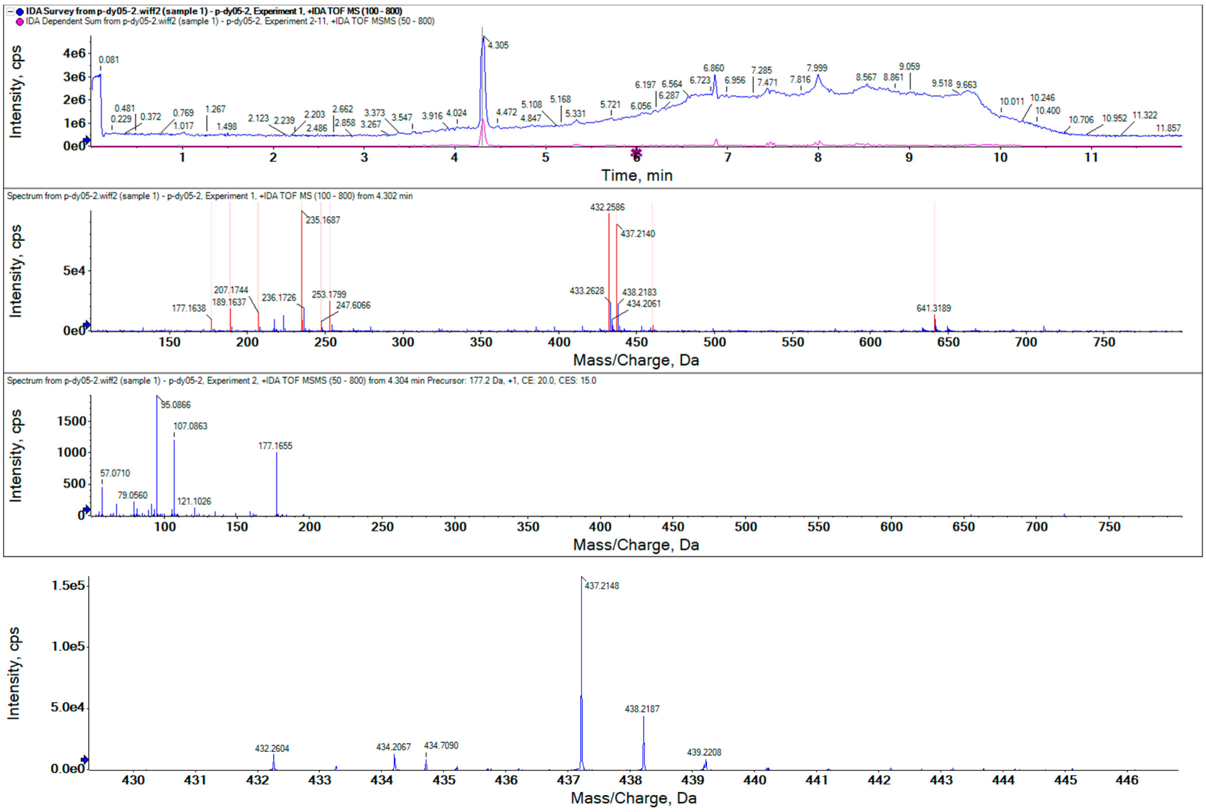

Figure S1. The HR-ESI-MS (MeOH) spectrum of **1**

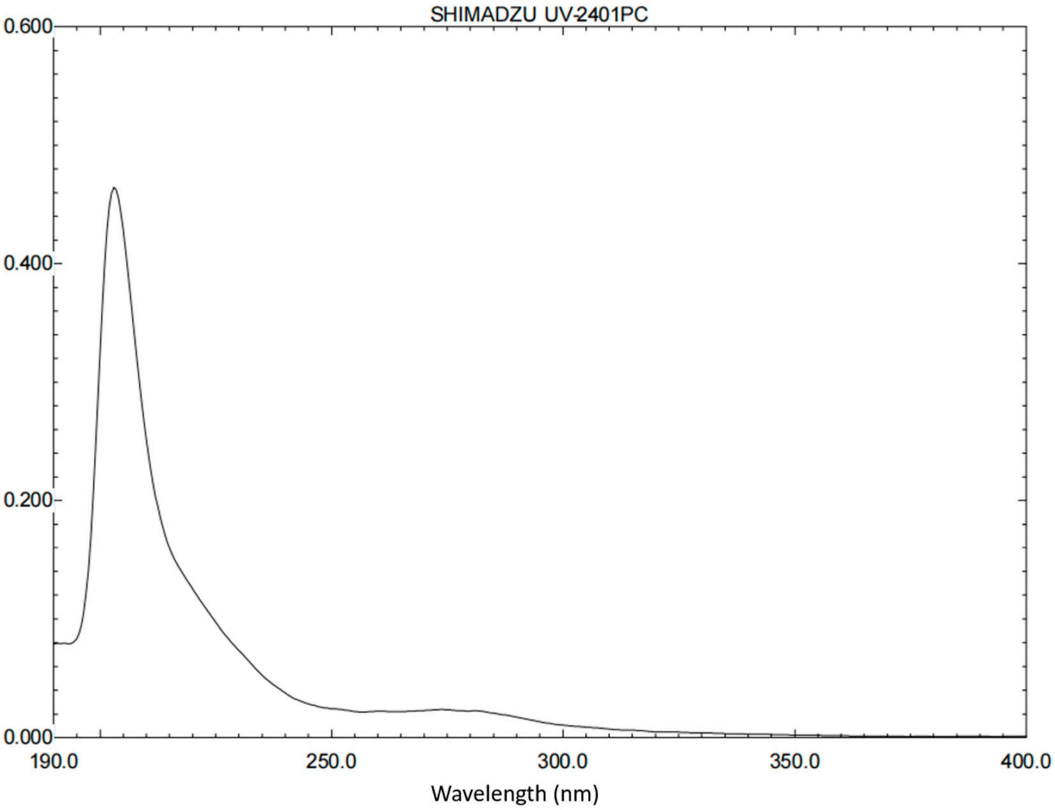

Figure S2. UV (MeOH) spectrum of **1**

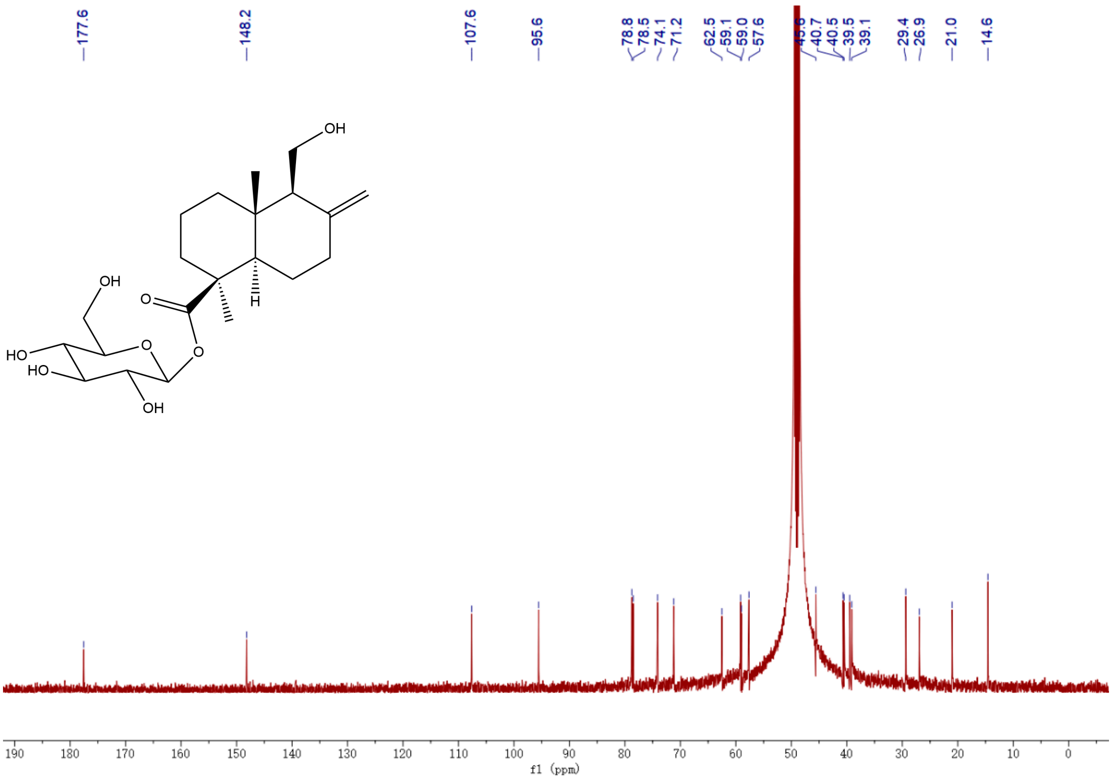

Figure S3. The ^{13}C NMR spectrum of **1** in CD_3OD (151 MHz)

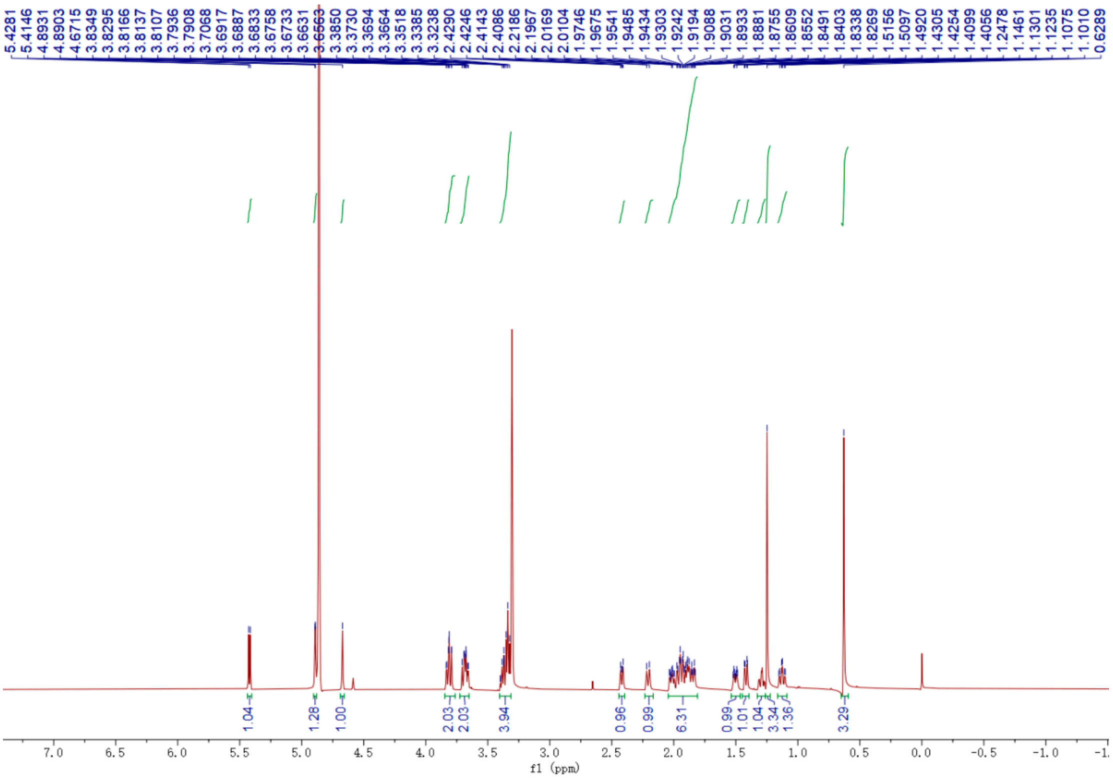

Figure S4. The ^{1}H NMR spectrum of **1** in CD_3OD (600 MHz)

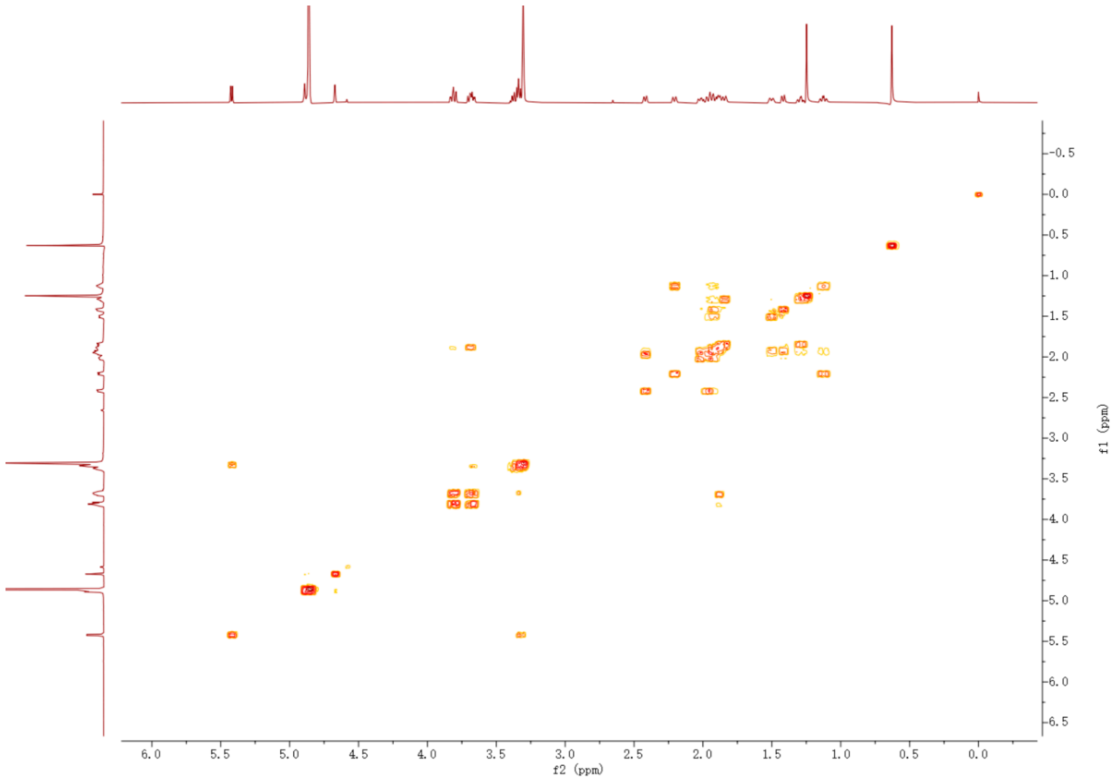

Figure S5. The ^{1}H-^{1}H COSY spectrum of **1** in CD_3OD

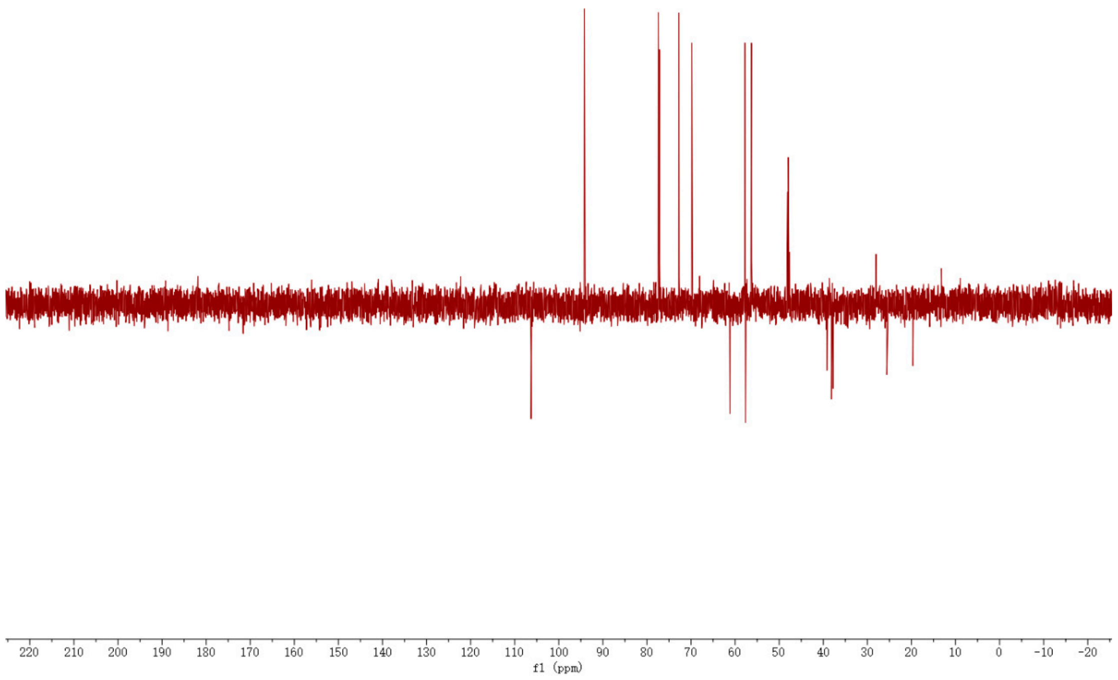

Figure S6. The DEPT spectrum of **1** in CD_3OD

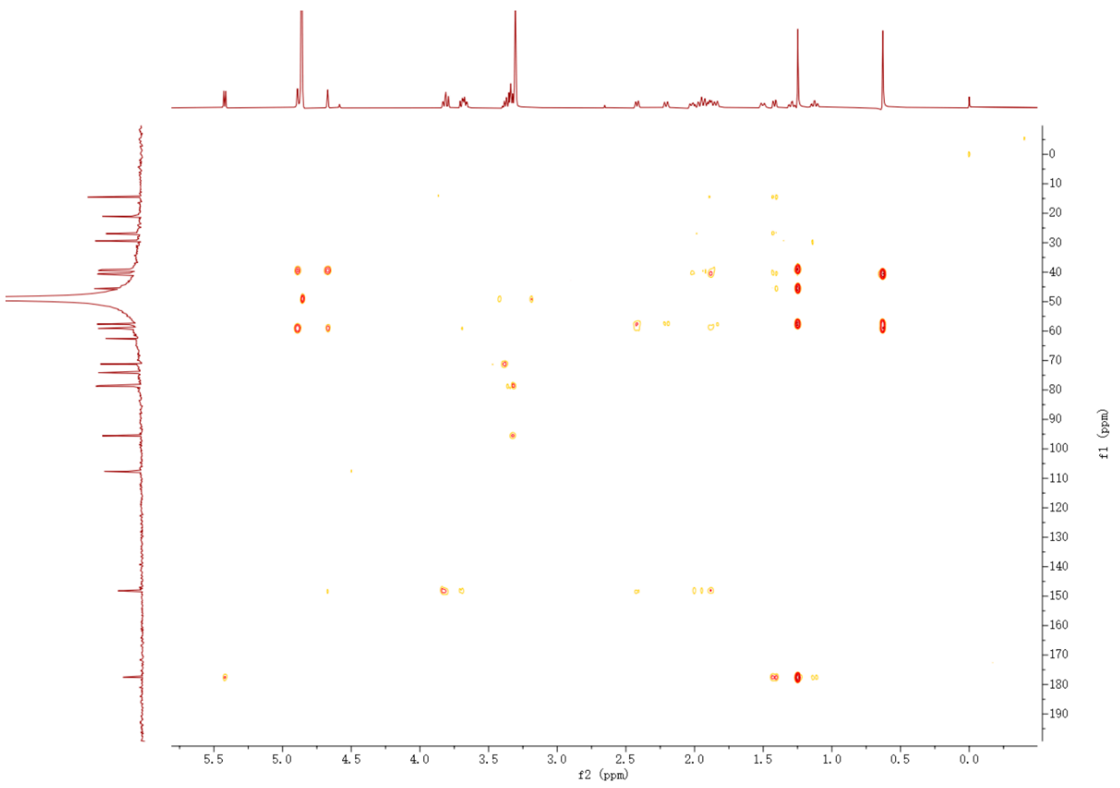

Figure S7. The HMBC spectrum of **1** in CD_3OD

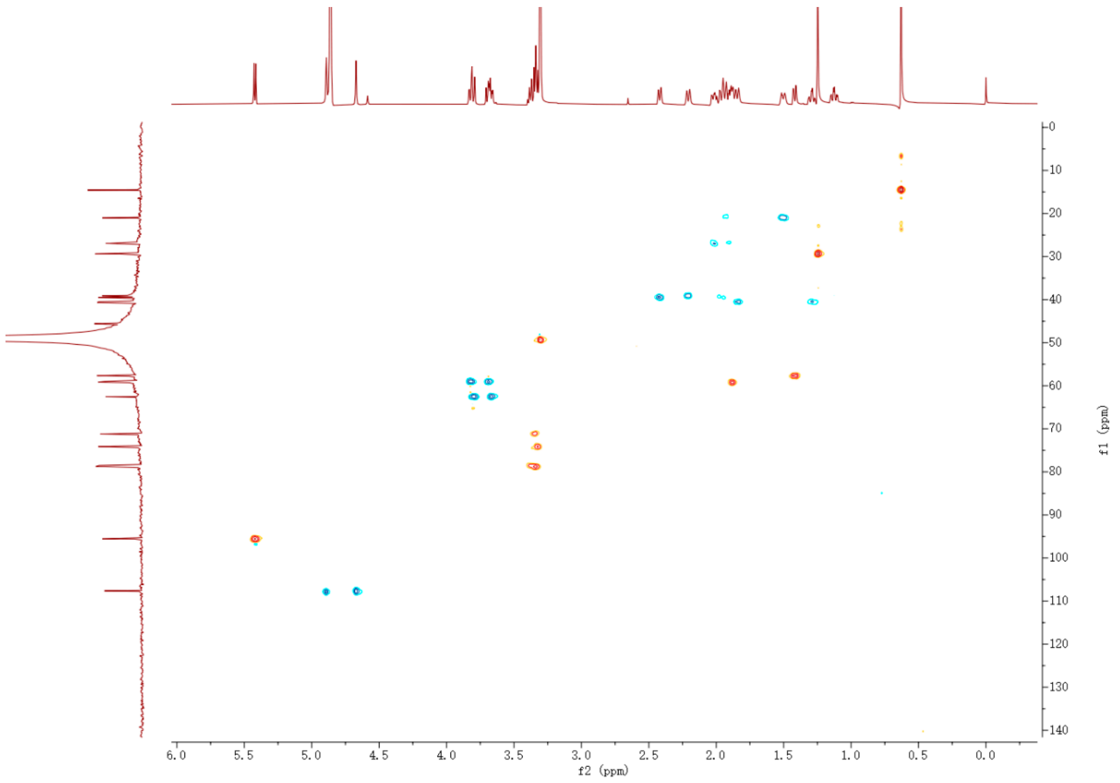

Figure S8. The HSQC spectrum of **1** in CD_3OD

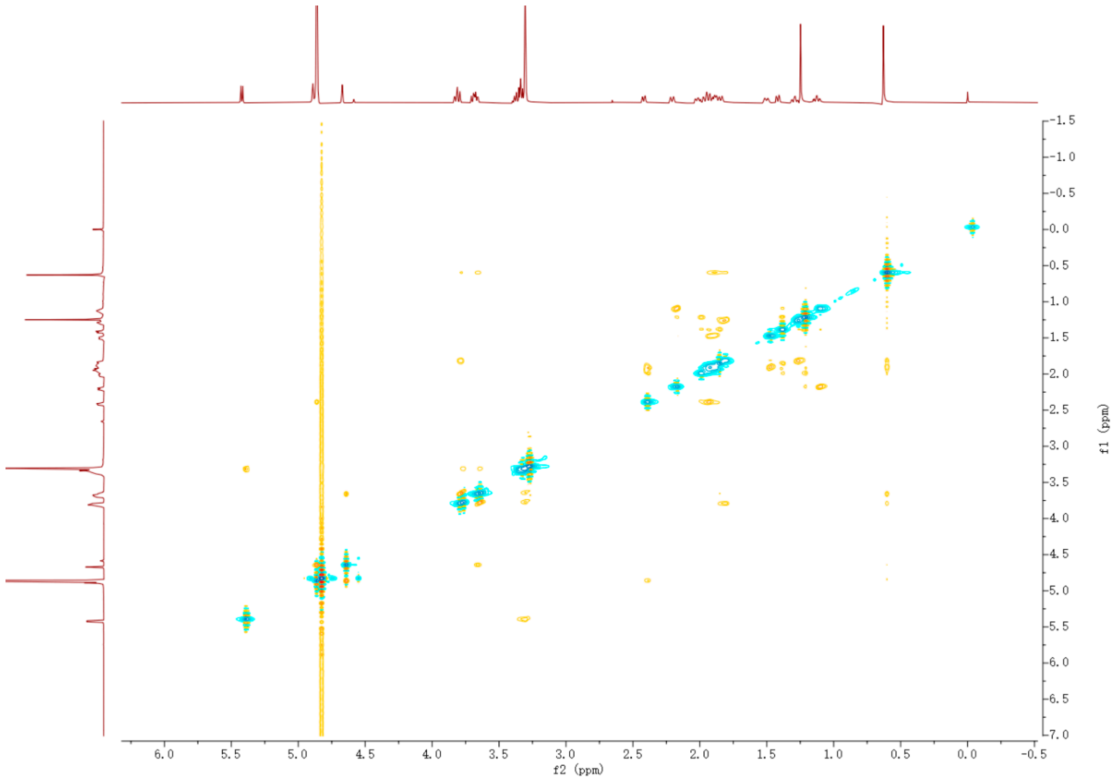

Figure S9. The NOESY spectrum of **1** in CD_3OD

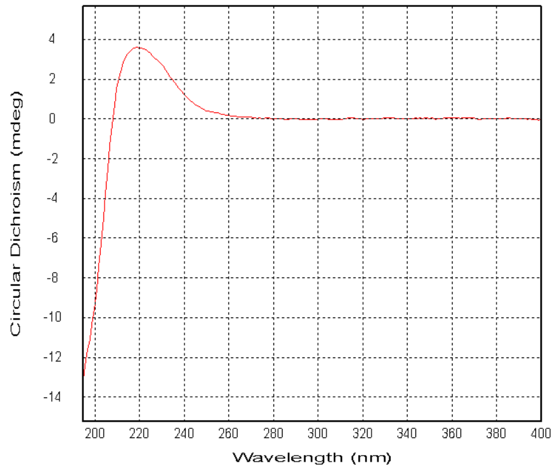

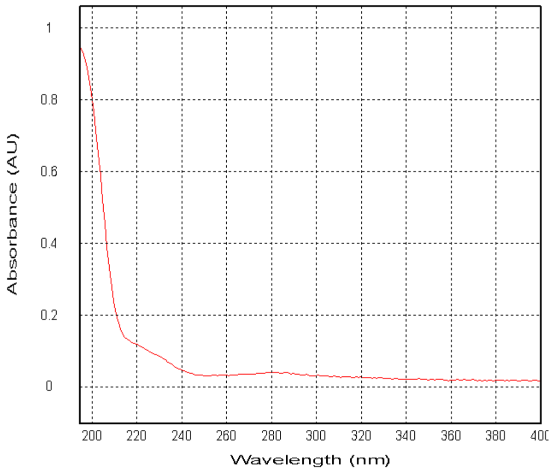

Figure S10. The CD spectrum of **1**

Table S1 ORD data of **1** (CD_3OD)

n	Average	Std.Dev	%RSD	Maximum	Minimum
5	14.40	0.55	3.81	15.00	14.00

S.No	Result	Scale	OR°Arc	WLG.nm	Lg.mm	Conc.g/100 mL	Temp.
1	15.00	SR	0.015	589	100.00	0.100	25.0
2	15.00	SR	0.015	589	100.00	0.100	25.0
3	14.00	SR	0.014	589	100.00	0.100	25.0
4	14.00	SR	0.014	589	100.00	0.100	25.0
5	14.00	SR	0.014	589	100.00	0.100	25.0

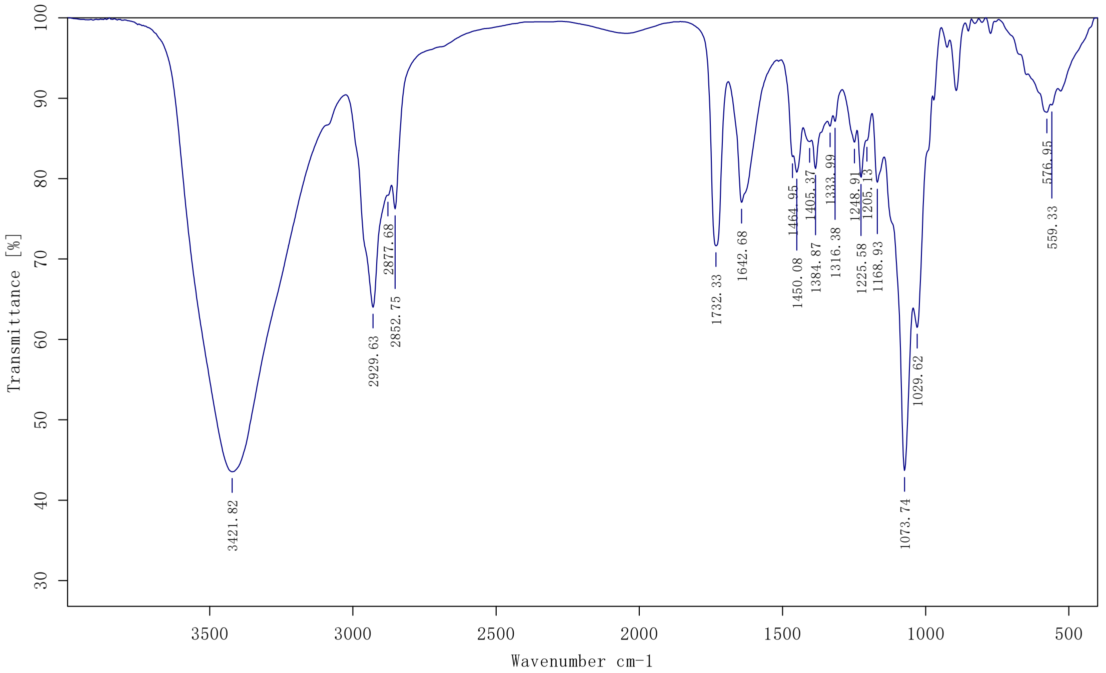

Sample Name: DY05
Sample Form: KBr
Path of File: E:\data
Date of Measurement: 2026/7/27

Resolution: 4
Aperture Setting: 6 mm
Number of Background Scans: 16
Number of Sample Scans: 16

Beamsplitter Setting: KBr
Source Setting: MIR
Instrument Type: BRUKER VERTEX 70
Soft Version: OPUS8.1

Figure S11. The IR spectrum of **1**

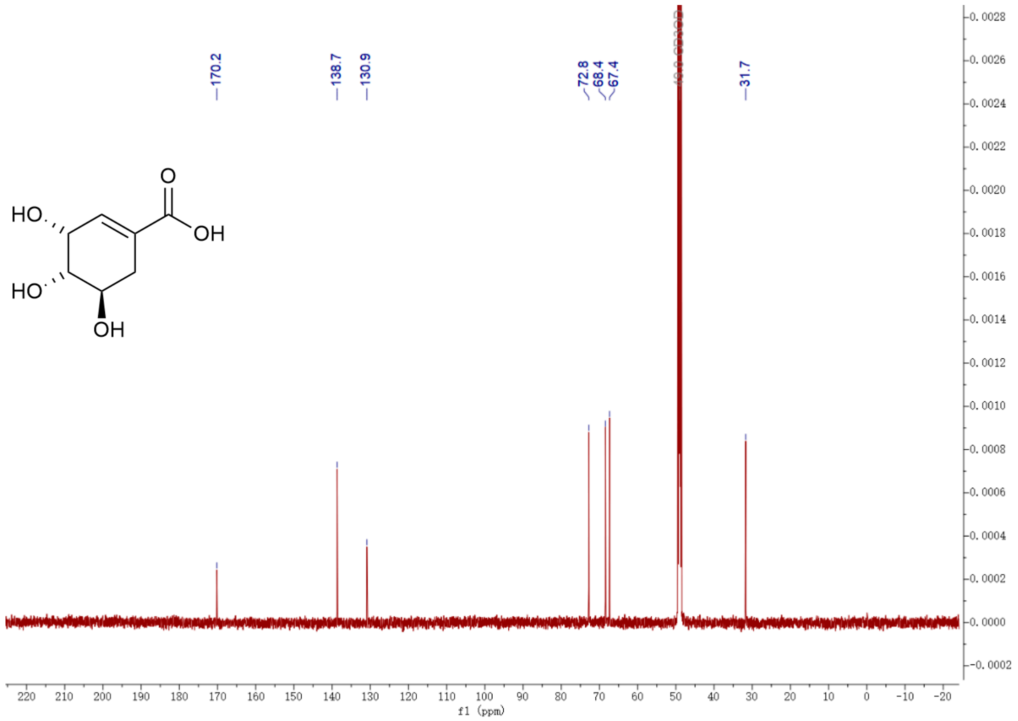

Figure S12. The ^{13}C NMR spectrum of **2** in CD_3OD (151 MHz)

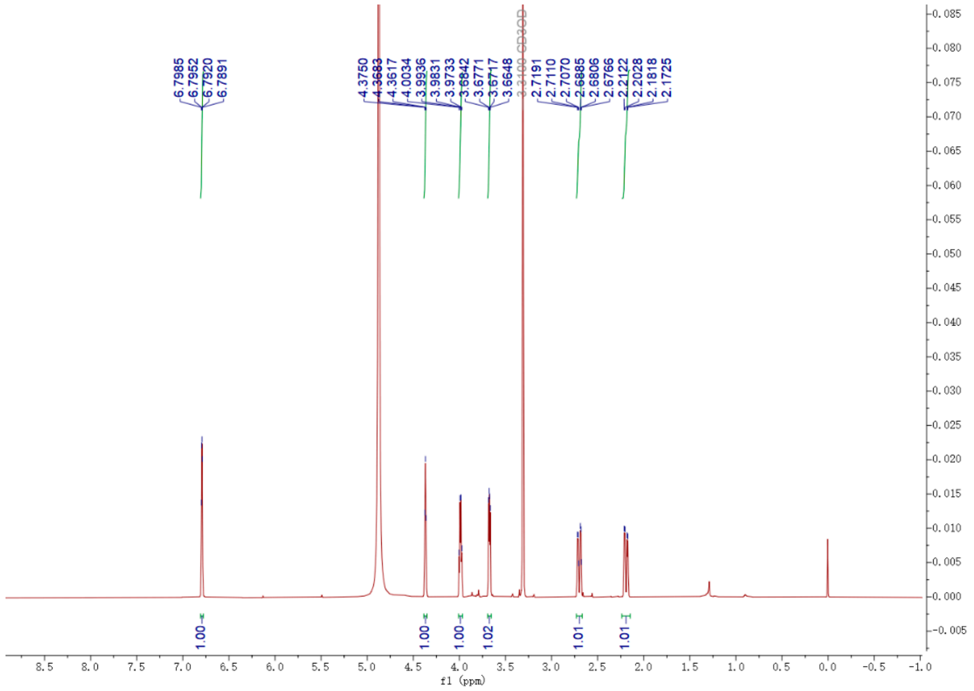

Figure S13. The ^{1}H NMR spectrum of **2** in CD_3OD (600 MHz)

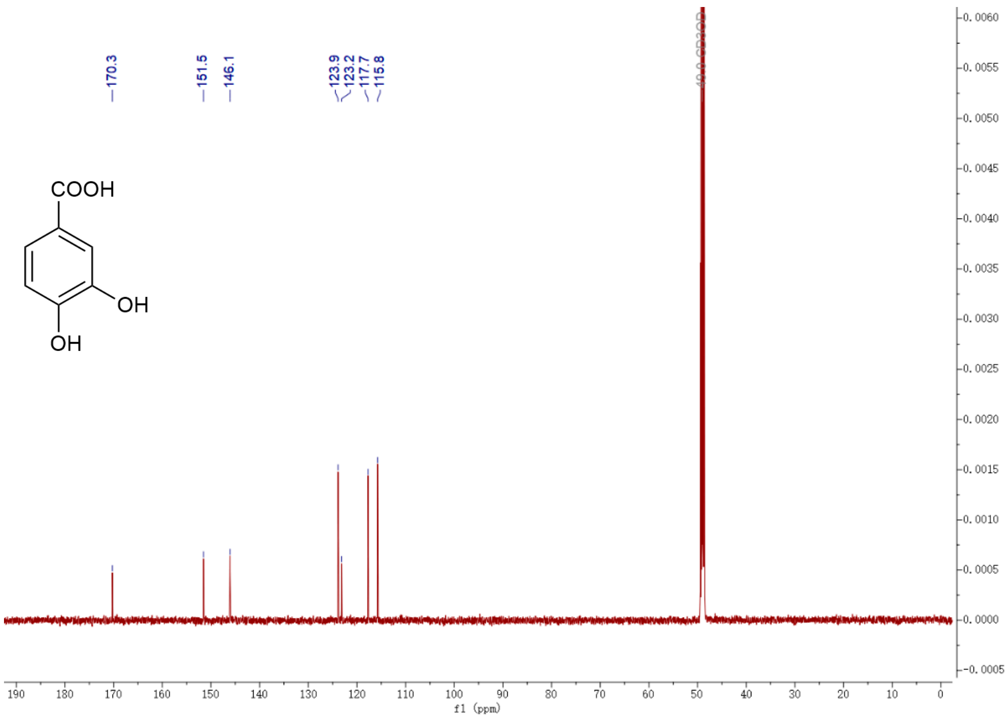

Figure S14. The ^{13}C NMR spectrum of **3** in CD_3OD (151 MHz)

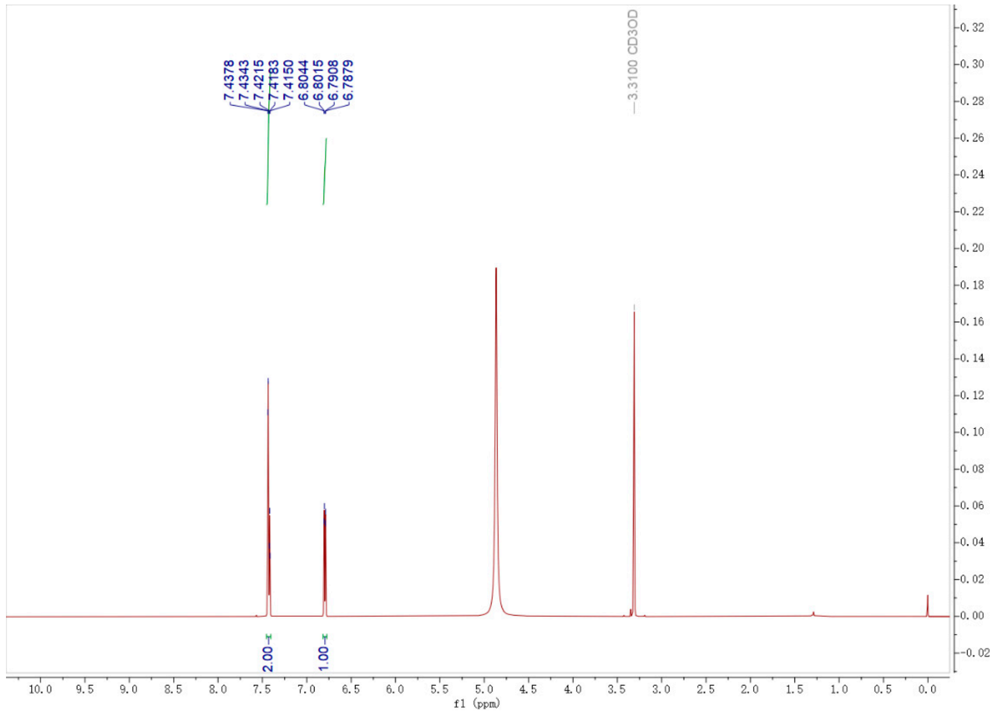

Figure S15. The ^{1}H NMR spectrum of **3** in CD_3OD (600 MHz)

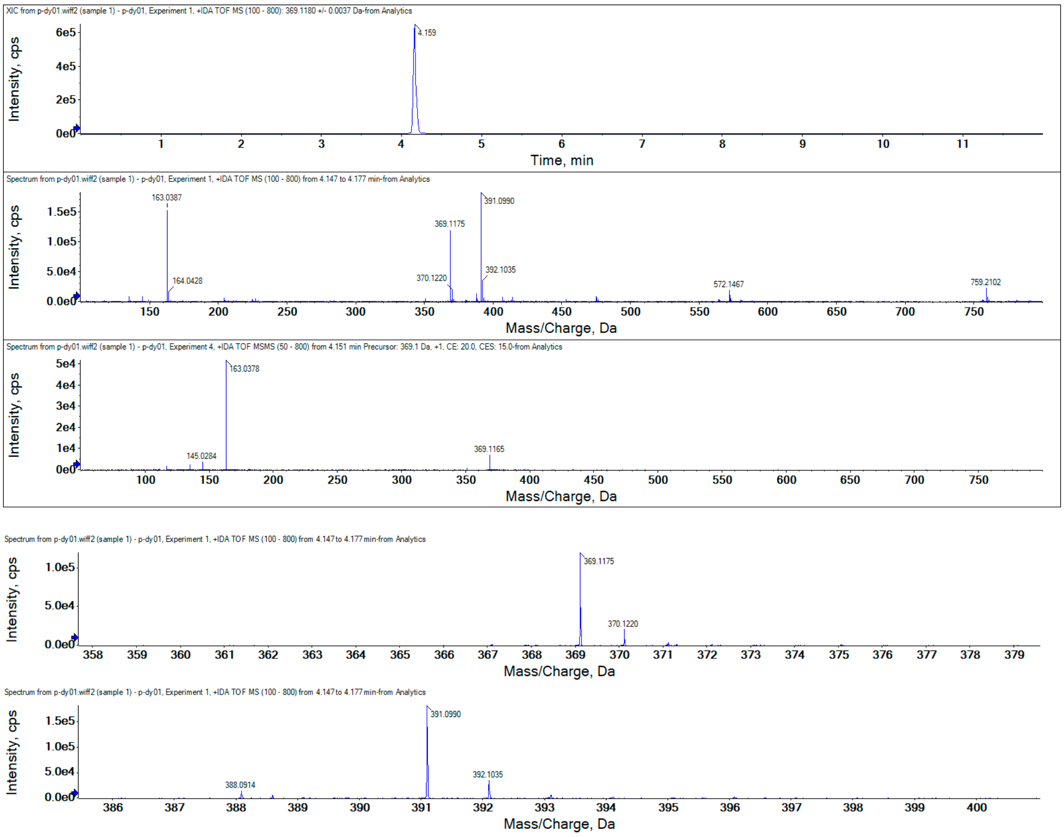

Figure S16. The HR-ESI-MS (MeOH) spectrum of **4**

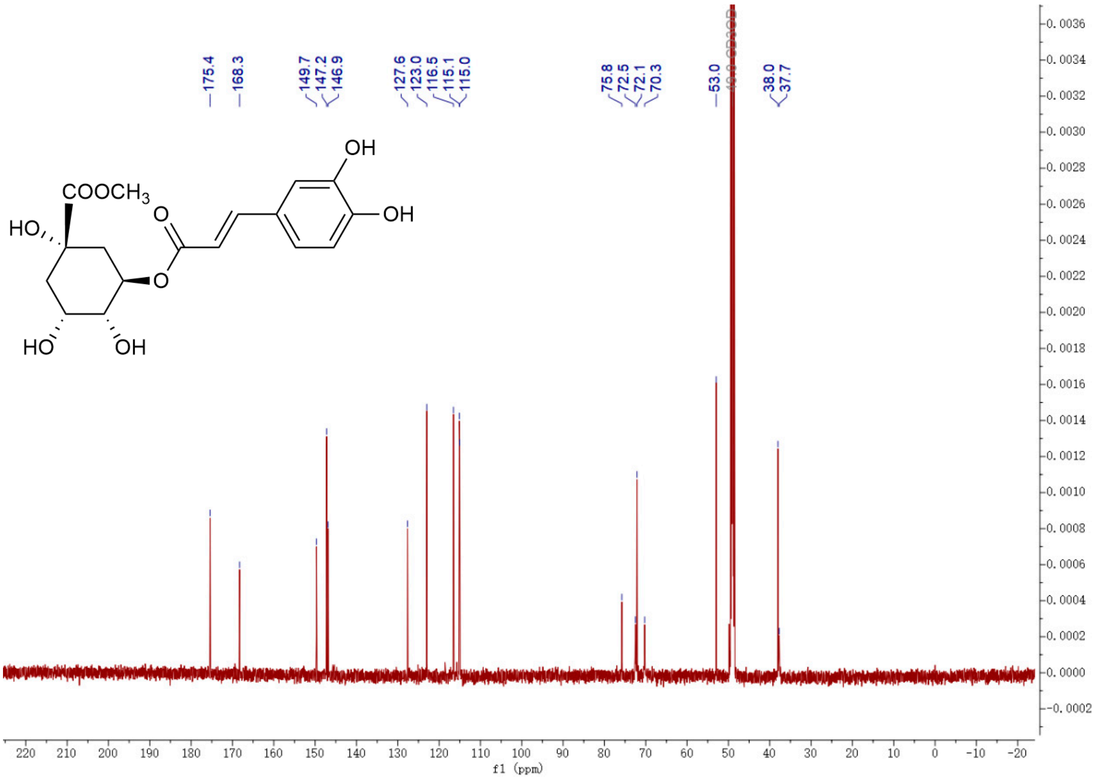

Figure S17. The ^{13}C NMR spectrum of **4** in CD_3OD (151 MHz)

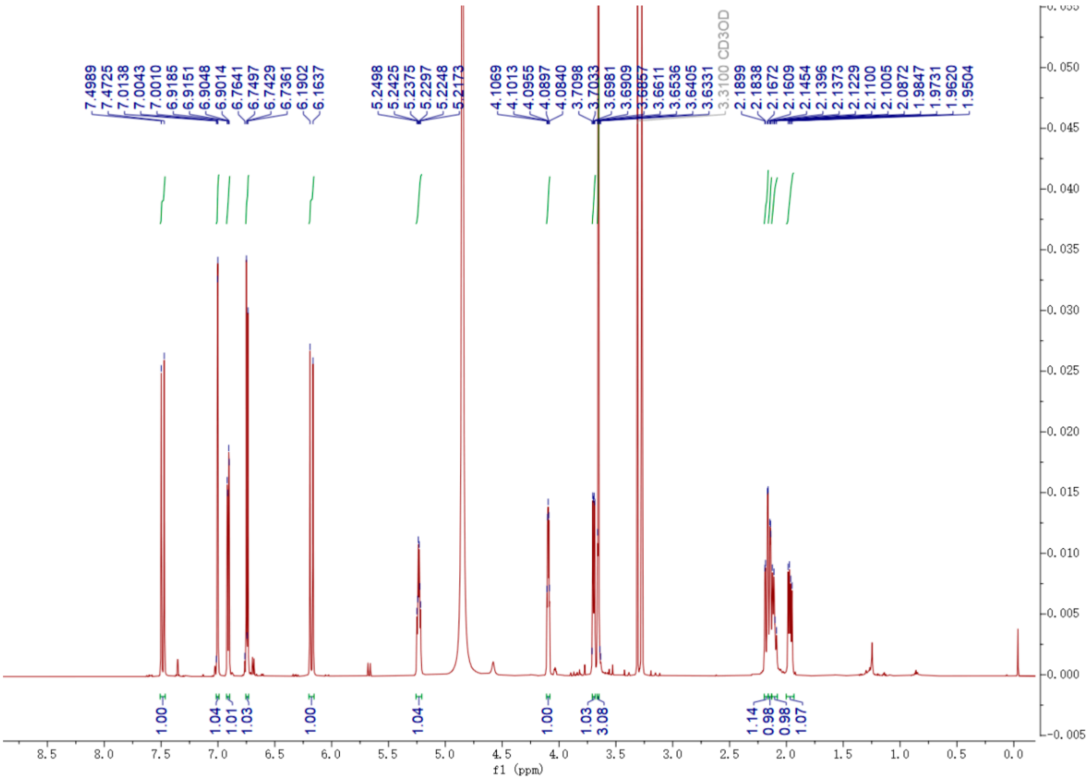

Figure S18. The ^{1}H NMR spectrum of **4** in CD_3OD (600 MHz)

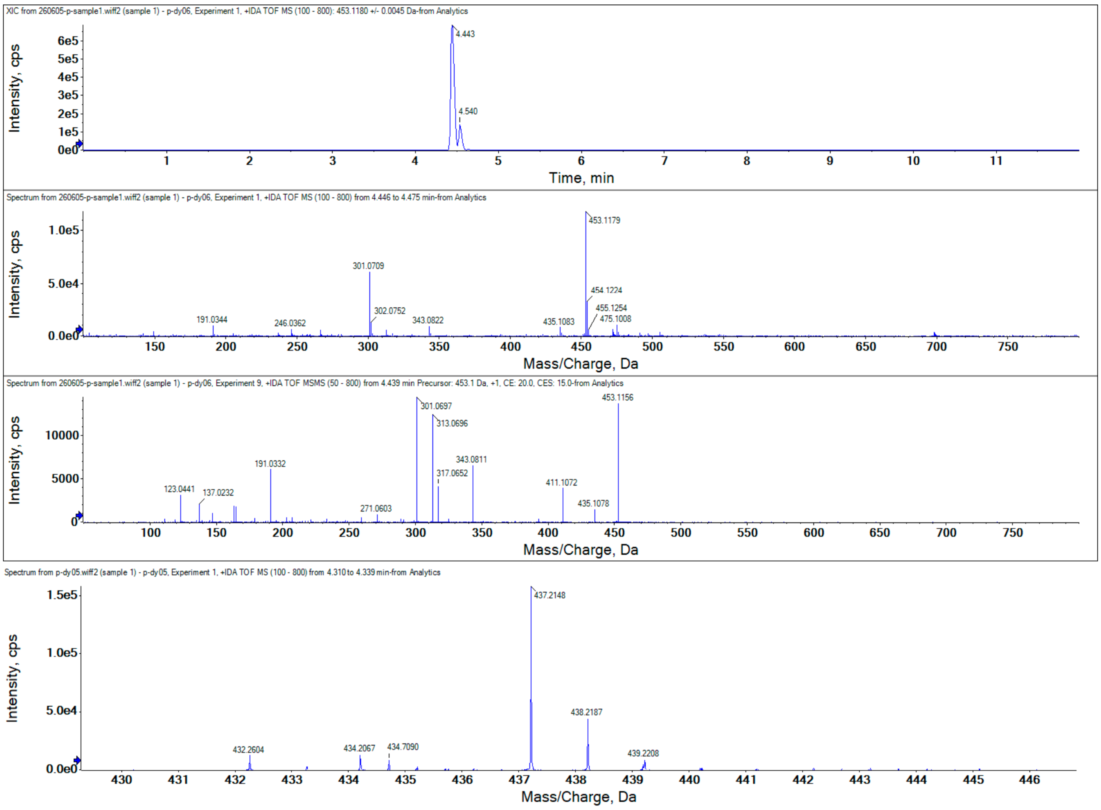

Figure S19. The HR-ESI-MS (MeOH) spectrum of **5**

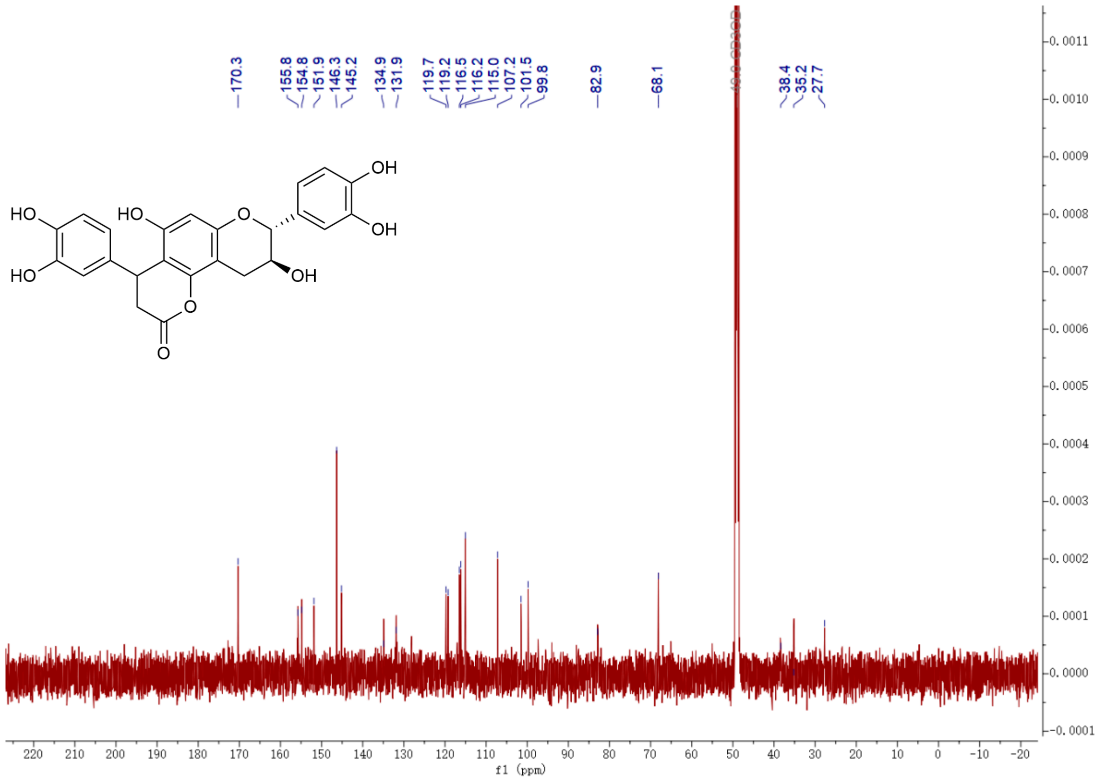

Figure S20. The ^{13}C NMR spectrum of **5** in CD_3OD (151 MHz)

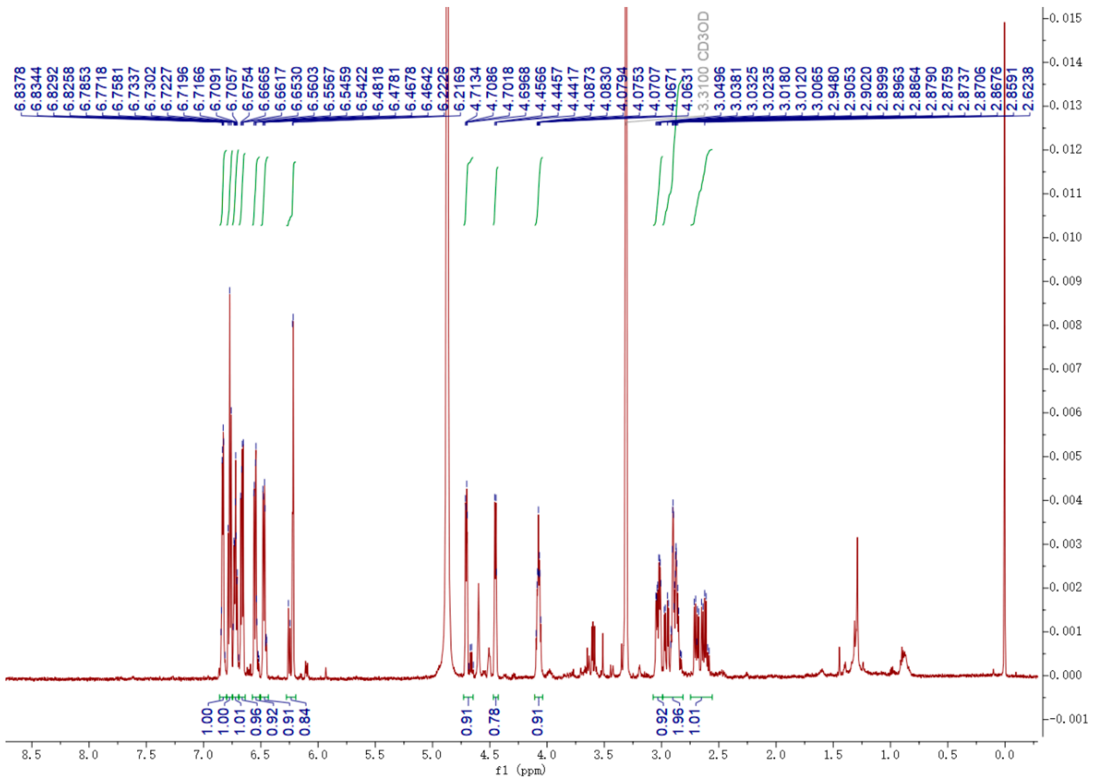

Figure S21. The ^{1}H NMR spectrum of **5** in CD_3OD (600 MHz)

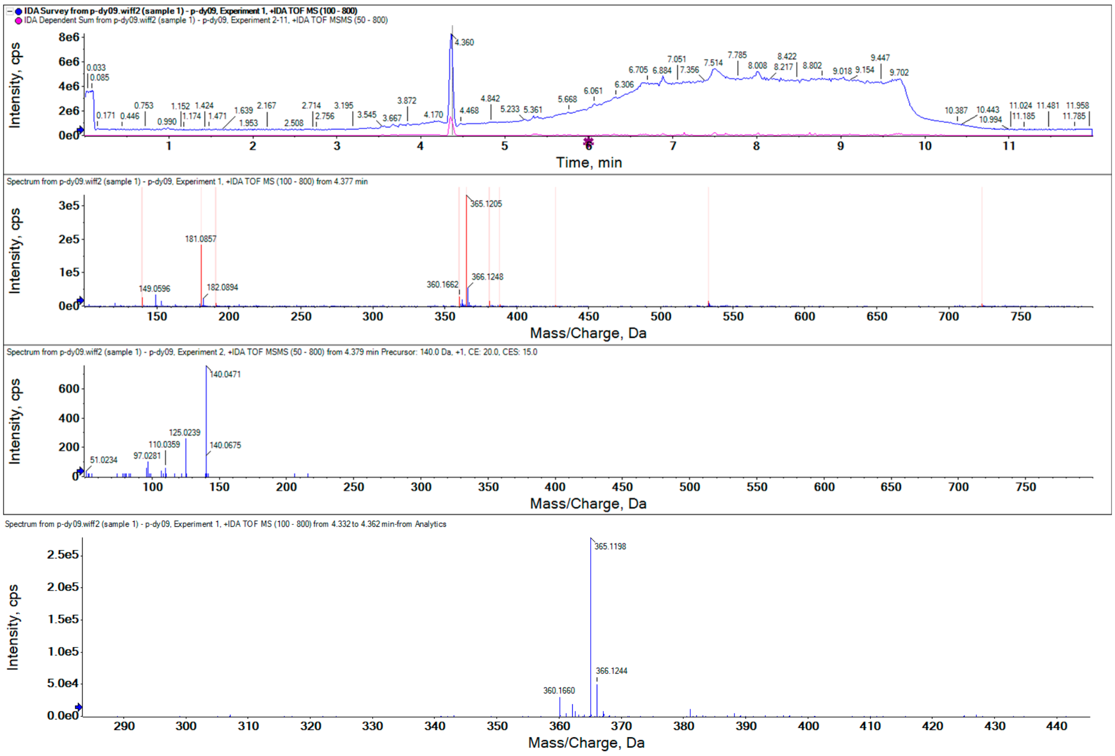

Figure S22. The HR-ESI-MS (MeOH) spectrum of **6**

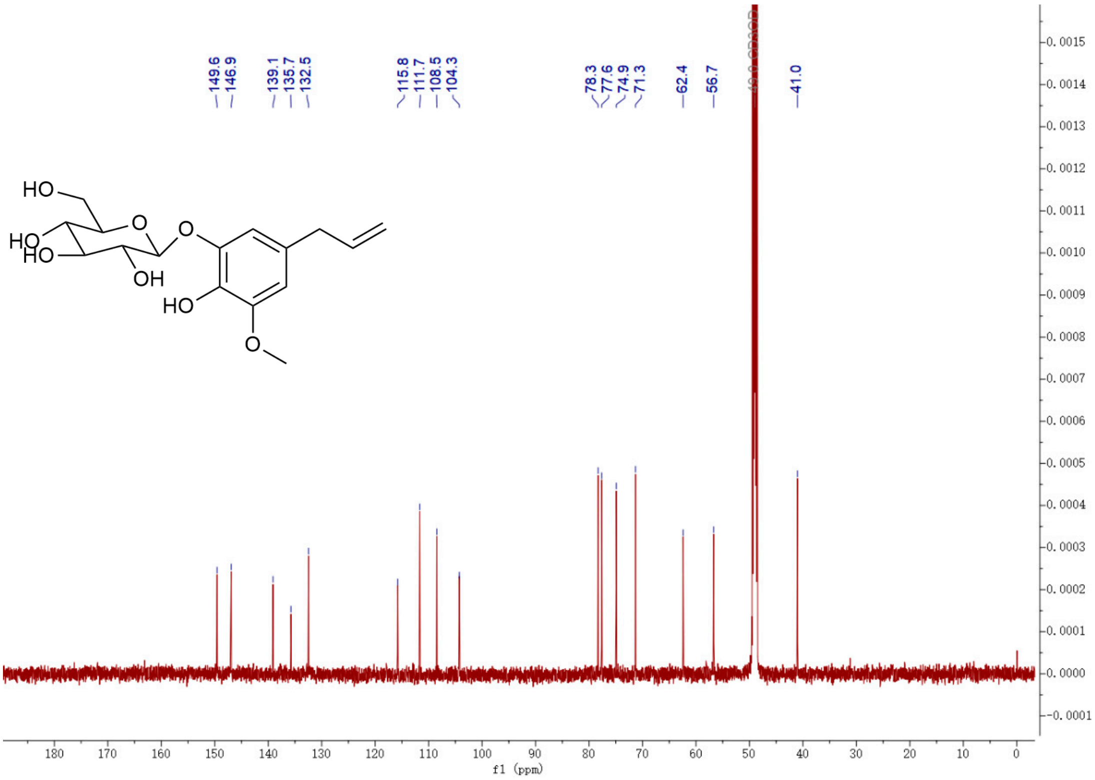

Figure S23. The ^{13}C NMR spectrum of **6** in CD_3OD (151 MHz)

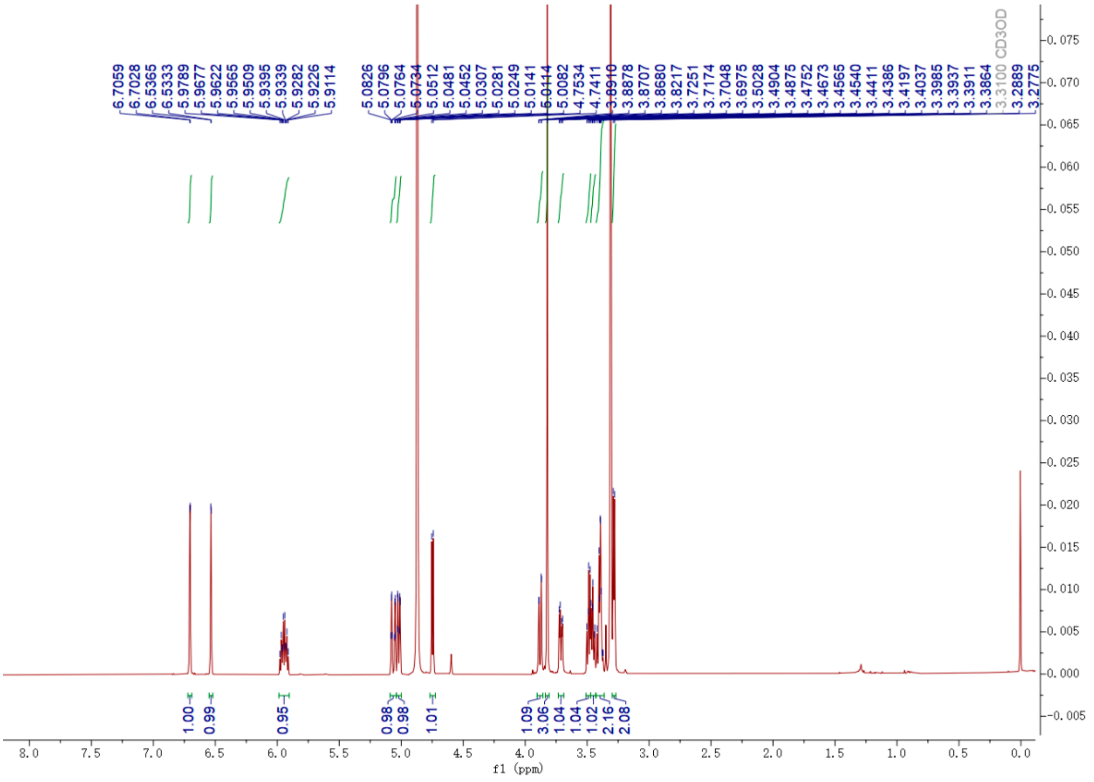

Figure S24. The ^{1}H NMR spectrum of **6** in CD_3OD (600 MHz)

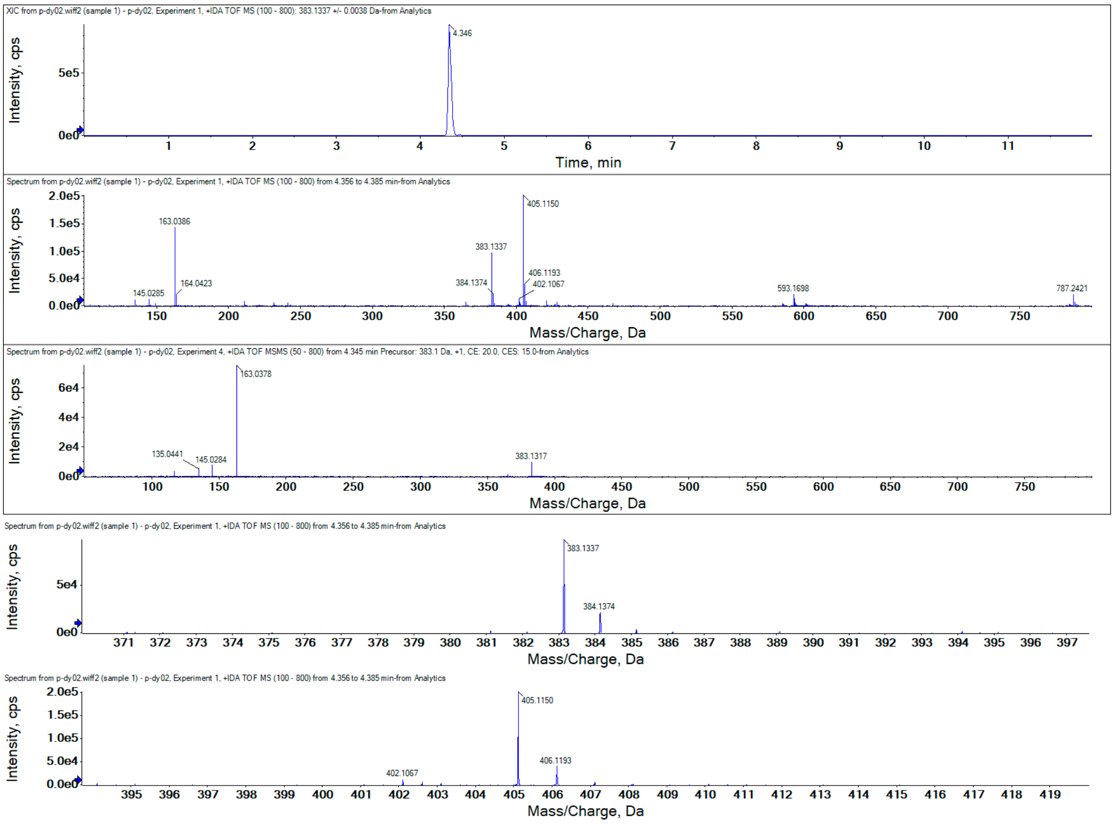

Figure S25. The HR-ESI-MS (MeOH) spectrum of **7**

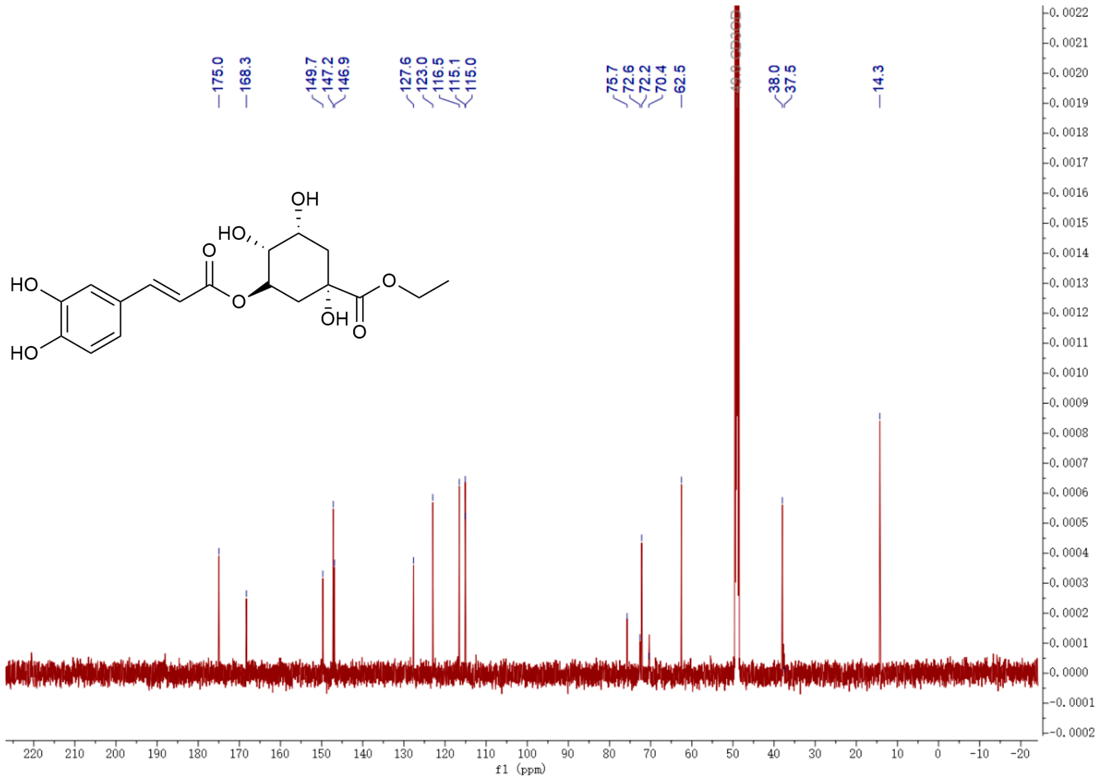

Figure S26. The ^{13}C NMR spectrum of **7** in CD_3OD (151 MHz)

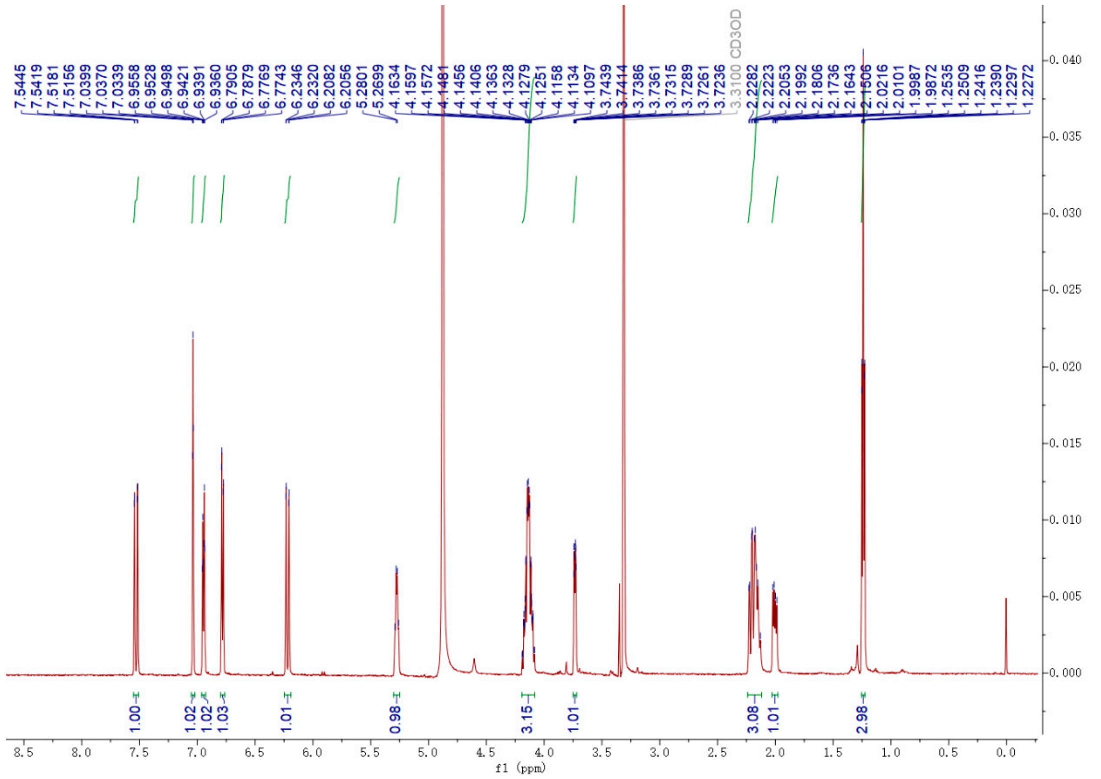

Figure S27. The ^{1}H NMR spectrum of **7** in CD_3OD (600 MHz)

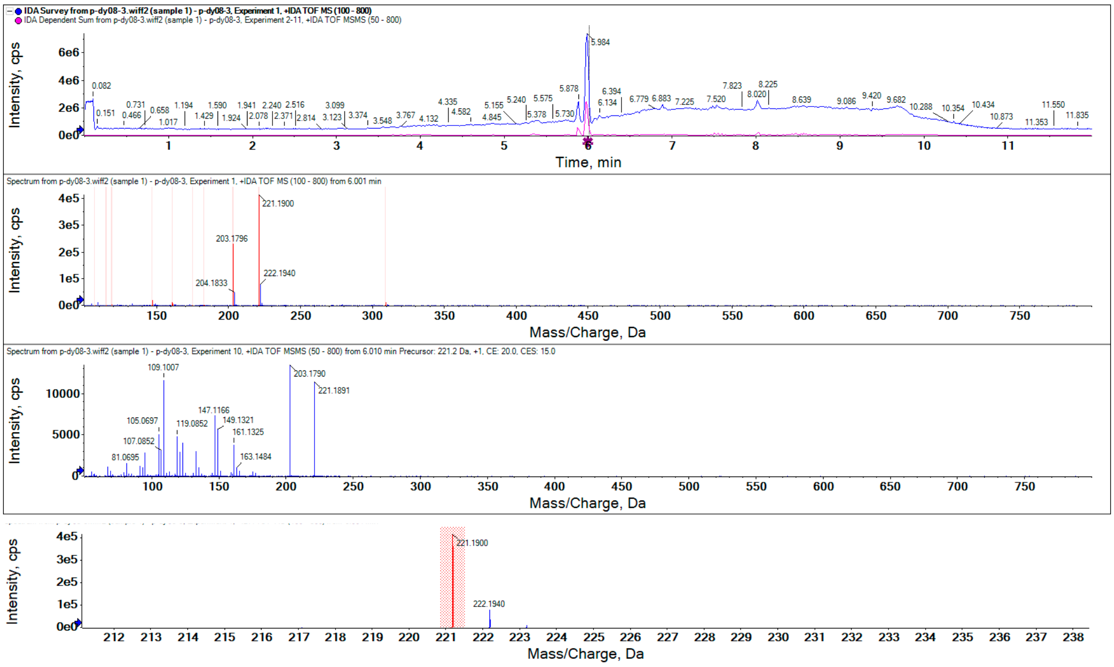

Figure S28. The HR-ESI-MS (MeOH) spectrum of **8**

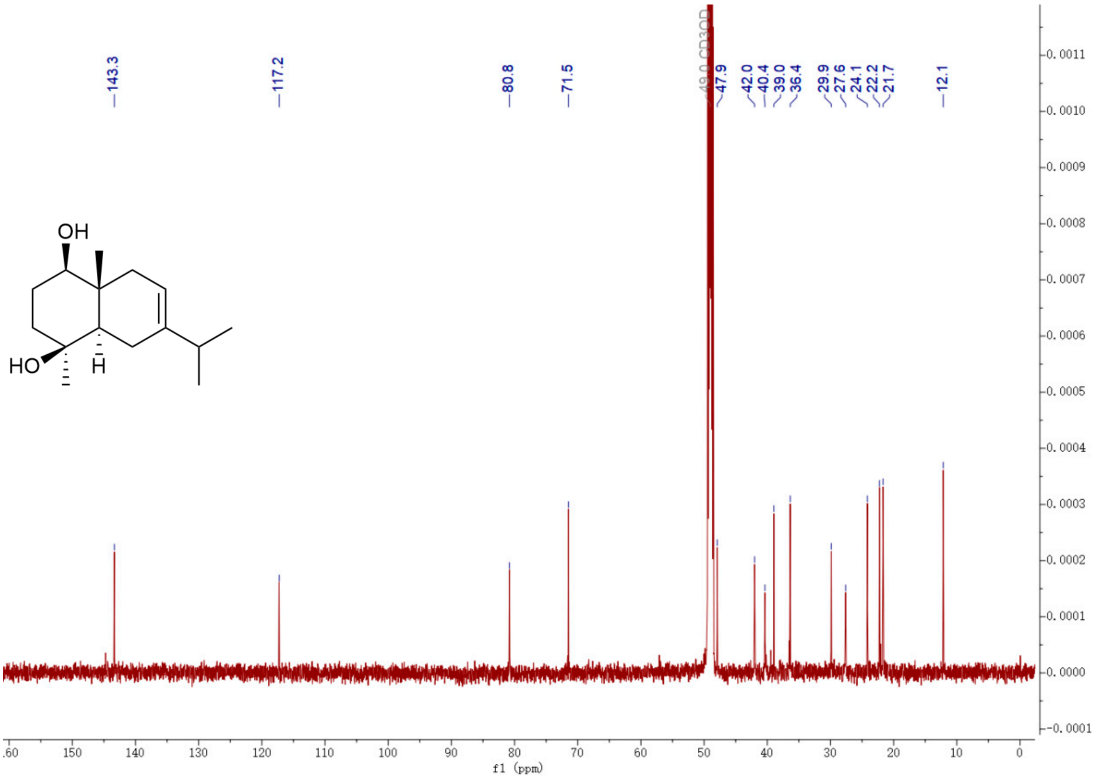

Figure S29. The ^{13}C NMR spectrum of **8** in CD_3OD (151 MHz)

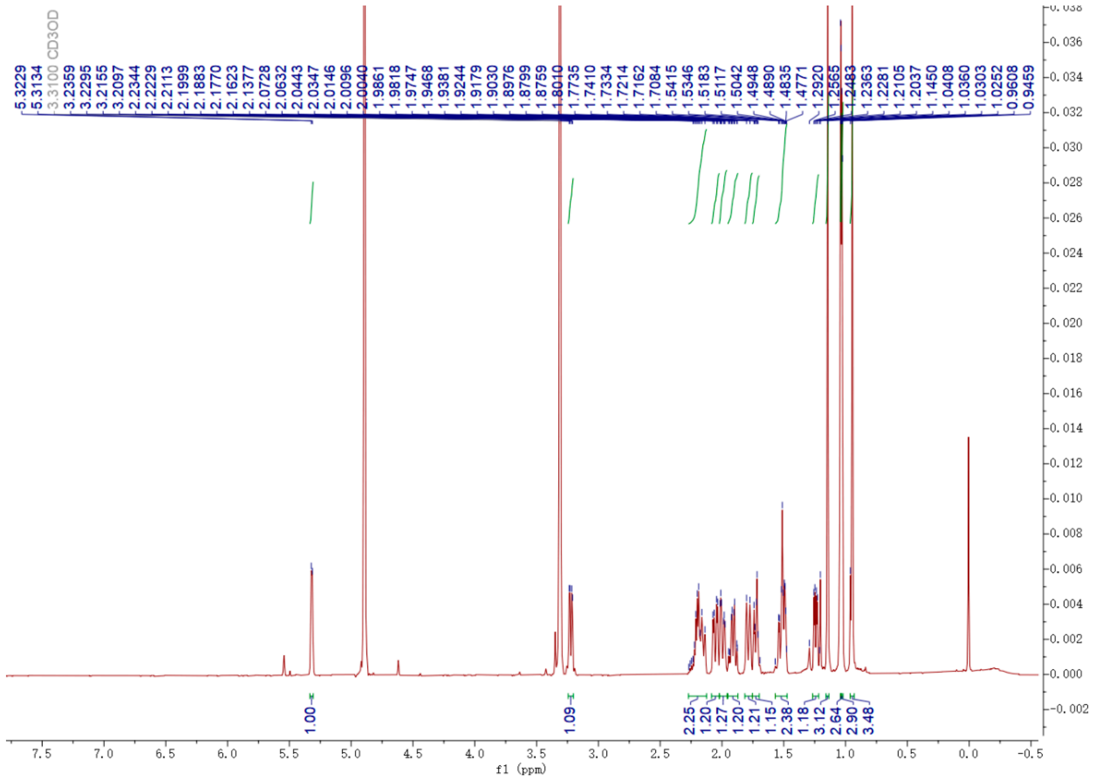

Figure S30. The ^{1}H NMR spectrum of **8** in CD_3OD (600 MHz)

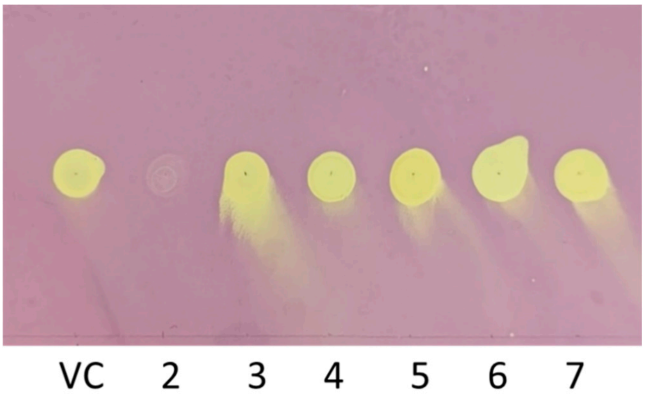

Figure S31. Antioxidant activity of compounds **2**-**7**

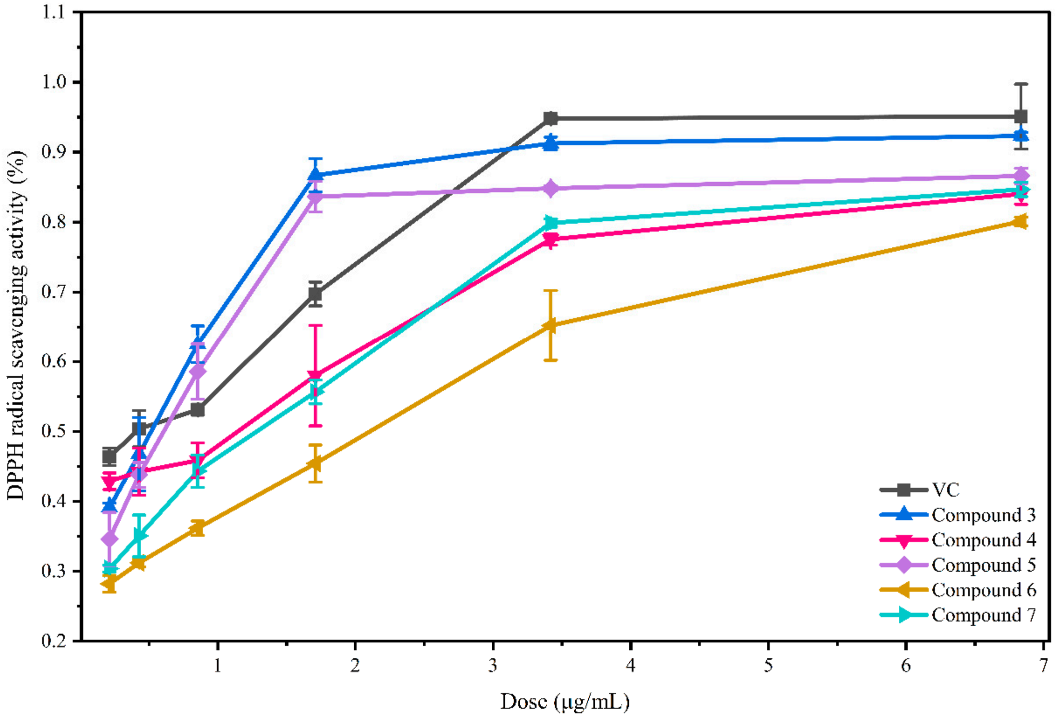

Figure S32. The DPPH radical scavenging activity of compounds **3**-**7**. Data are presented as mean ± SD (n = 3)

Table S2 DPPH radical scavenging activity of compounds **2**–**7** at different concentrations

Compound **2** concentration (μg/mL)	13.672	27.344	54.688	109.375	218.750	437.500	IC_{50} (μg/mL)
DPPH radical scavenging rate (%)	35.43 ± 2.98[d]	36.66 ± 2.52[d]	38.76 ± 3.00[d]	49.94 ± 2.18[c]	65.95 ± 0.92[b]	76.10 ± 1.91[a]	74.337

Sample	DPPH radical scavenging rate (%) at different concentrations (μg/mL)						IC_{50}
	0.213	0.428	0.854	1.710	3.418	6.836	(μg/mL)
VC	46.37 ± 1,26[d]	50.38 ± 2.59[cd]	53.11 ± 0.79[c]	69.72 ± 1.69[b]	94.81 ± 0.38[a]	95.09 ± 4.65[a]	0.402
Compound **3**	39.12 ± 0.63[d]	46.77 ± 5.23[c]	62.50 ± 2.65[b]	86.68 ± 2.38[a]	91.24 ± 0.91[a]	92.31 ± 0.54[a]	0.401
Compound **4**	42.89 ± 1.17[c]	44.23 ± 3.35[c]	45.86 ± 2.51[c]	58.03 ± 7.23[b]	77.53 ± 0.78[a]	84.03 ± 1.51[a]	0.610
Compound **5**	34.64 ± 3.79[d]	43.80 ± 1.78[c]	58.58 ± 3.96[b]	83.63 ± 2.21[a]	84.82 ± 0.34[a]	86.60 ± 1.10[a]	0.481
Compound **6**	28.22 ± 1.21[e]	31.23 ± 0.56[de]	36.19 ± 1.01[d]	45.42 ± 2.65[c]	65.20 ± 5.01[b]	80.10 ± 0.61[a]	1.414
Compound **7**	30.40 ± 0.50[d]	35.08 ± 2.99[d]	44.35 ± 2.31[c]	55.65 ± 1.69[b]	79.87 ± 0.60[a]	84.66 ± 1.02[a]	0.885

Note: Data of DPPH radical scavenging rate are presented as mean ± SD ($n = 3$). Statistical analysis was performed using one-way ANOVA followed by Tukey's post hoc test. Different lowercase letters within the same compound indicate significant differences ($p < 0.05$).

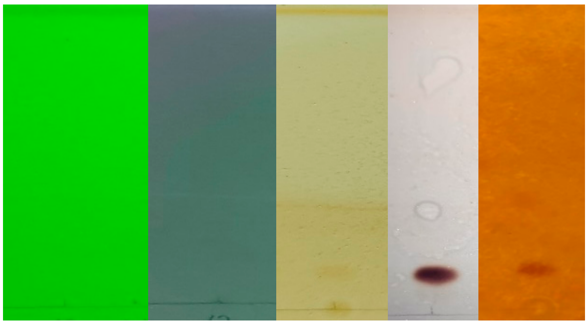

Figure S33. TLC analysis of compound **1** ($CHCl_3$:MeOH=7:1)
(Note: From left to right, the colors are UV 254 nm, UV 365 nm, iodine, concentrated sulfuric acid, and potassium bismuth iodide)

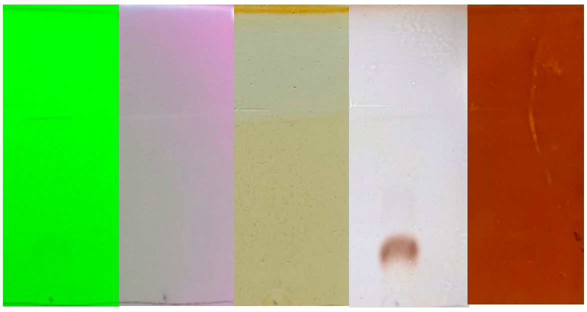

Figure S34. TLC analysis of compound **2** ($CHCl_3$:MeOH=4:1)
(Note: From left to right, the colors are UV 254 nm, UV 365 nm, iodine, concentrated sulfuric acid, and potassium bismuth iodide)

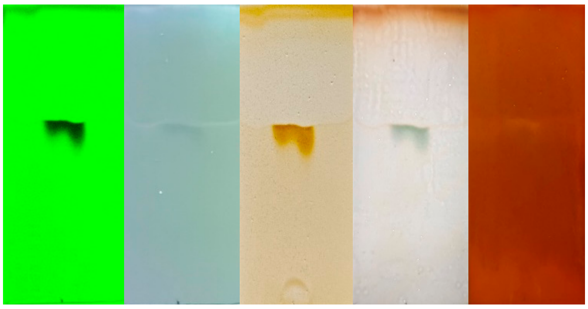

Figure S35. TLC analysis of compound **3** ($CHCl_3$:MeOH=4:1)
(Note: From left to right, the colors are UV 254 nm, UV 365 nm, iodine, concentrated sulfuric acid, and potassium bismuth iodide)

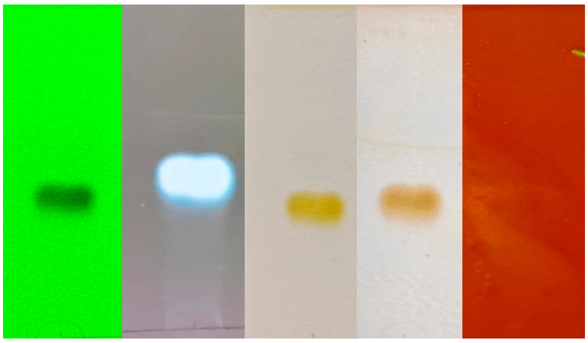

Figure S36. TLC analysis of compound **4** ($CHCl_3$:MeOH=5:1)
(Note: From left to right, the colors are UV 254 nm, UV 365 nm, iodine, concentrated sulfuric acid, and potassium bismuth iodide)

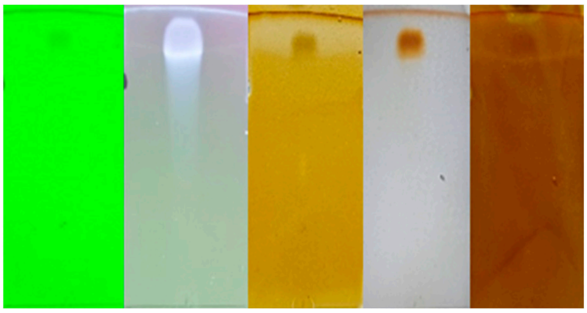

Figure S37. TLC analysis of compound **5** ($CHCl_3$:MeOH=3:1)
(Note: From left to right, the colors are UV 254 nm, UV 365 nm, iodine, concentrated sulfuric acid, and potassium bismuth iodide)

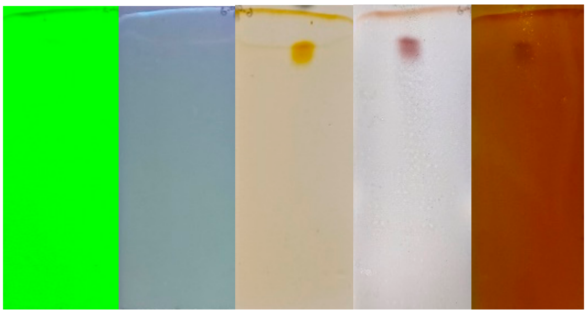

Figure S38. TLC analysis of compound **6** ($CHCl_3$:MeOH=3:1)
(Note: From left to right, the colors are UV 254 nm, UV 365 nm, iodine, concentrated sulfuric acid, and potassium bismuth iodide)

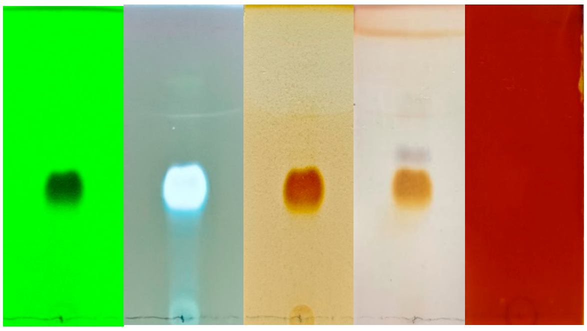

Figure S39. TLC analysis of compound **7** ($CHCl_3$:MeOH=7:1)

(Note: From left to right, the colors are UV 254 nm, UV 365 nm, iodine, concentrated sulfuric acid, and potassium bismuth iodide)

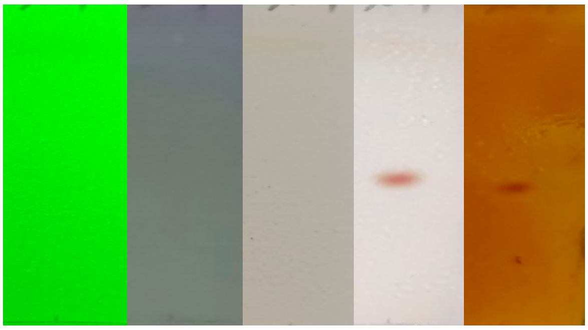

Figure S40. TLC analysis of compound **8** ($CHCl_3$:MeOH=10:1)

(Note: From left to right, the colors are UV 254 nm, UV 365 nm, iodine, concentrated sulfuric acid, and potassium bismuth iodide)

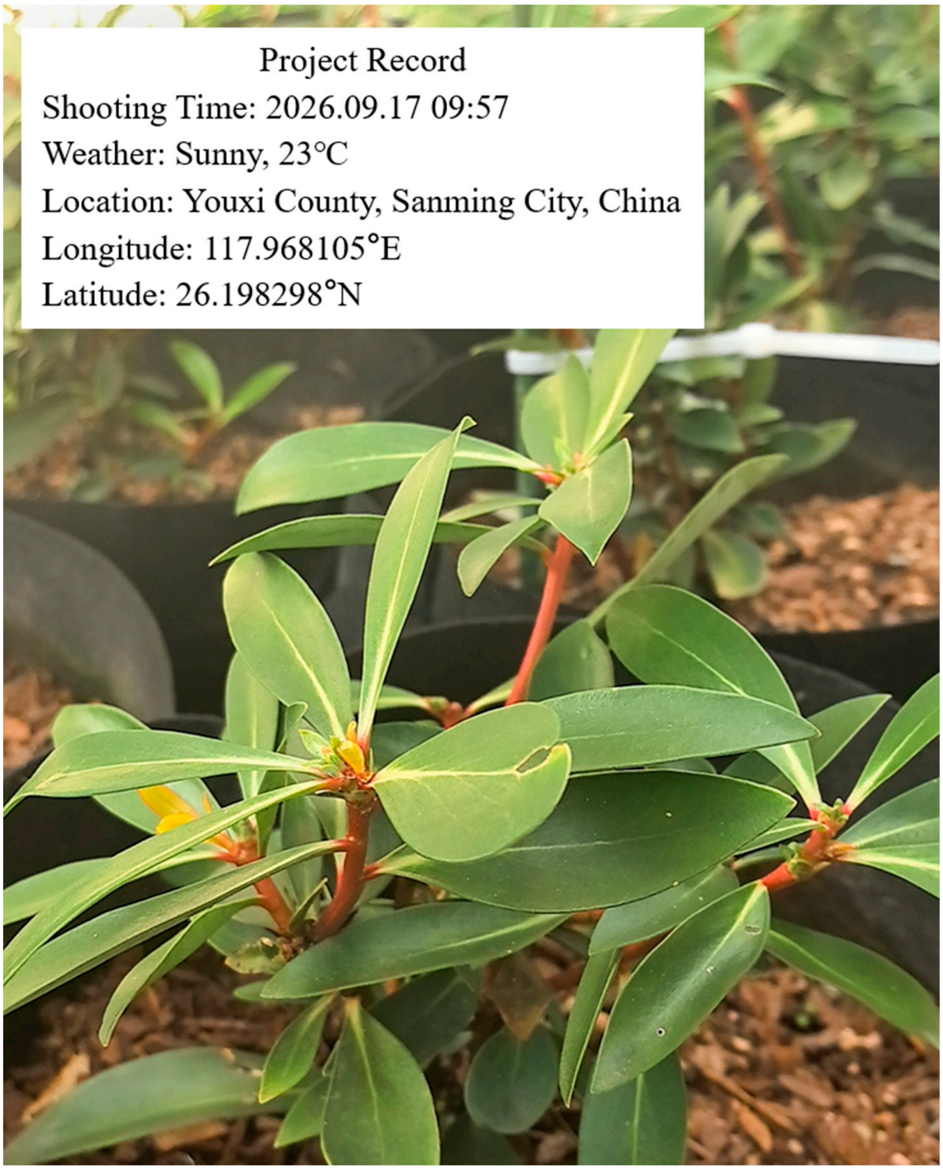

Figure S41. Morphology of the *T. lanceolata* used as plant material in this study
